# Enhanced ferroptosis sensitivity promotes the formation of highly myopic cataract via the DDR2-Hippo pathway

**DOI:** 10.1038/s41419-025-07384-8

**Published:** 2025-02-03

**Authors:** Dongling Guo, Yu Du, Xin Liu, Dan Li, Ling Wei, Xiangjia Zhu

**Affiliations:** 1 Eye Institute and Department of Ophthalmology, Eye & ENT Hospital, Fudan University, Shanghai, China; 2 Key Laboratory of Myopia and Related Eye Diseases, NHC; Key Laboratory of Myopia and Related Eye Diseases, Chinese Academy of Medical Sciences, Shanghai, China; 3 Shanghai Key Laboratory of Visual Impairment and Restoration, Shanghai, China; 4 State Key Laboratory of Medical Neurobiology, Fudan University, Shanghai, China

**Keywords:** Diseases, Cell biology

## Abstract

Highly myopic cataract (HMC) is a leading cause of blindness among the working-age individuals, with its pathogenesis poorly understood. This study aimed to elucidate the role of ferroptosis in HMC development as well as the underlying mechanisms. In HMC lens epithelia, levels of Fe^2+^ and lipid peroxidation were found elevated, with increased vulnerability towards ferroptosis as revealed by transmission electron microscopy. Mechanistically, RNA sequencing of HMC lens epithelial samples identified up-regulated expression of discoidin domain receptor tyrosine kinase 2 (DDR2) as a key factor, which could enhance ferroptosis sensitivity via the Src-Hippo pathway. Specifically, DDR2 interacted with Src kinase, leading to the nuclear translocation of homologous transcriptional regulators (yes-associated protein 1 [YAP1] and WW domain containing transcription regulator 1 [WWTR1]) of the Hippo pathway, which altered the expression level of ferroptosis-related genes. Notably, highly myopic eyes of mice exhibited higher sensitivity to RSL3, a ferroptosis inducer, manifested as more severe nuclear lens opacities both in vitro and in vivo compared with the contralateral control eyes, which could be alleviated by inhibitors of either ferroptosis or DDR2. Altogether, these findings highlighted the role of DDR2 in mediating ferroptosis in HMC formation, providing a novel insight for therapeutic interventions.

## Introduction

The prevalence of high myopia is increasing globally and has become a worldwide health challenge [1, 2]. Highly myopic cataract (HMC), the most common complication of high myopia, manifests approximately a decade earlier than the age-related cataract (ARC) [3], significantly impacting individuals’ quality of life during their working years. Surgical intervention for HMC is particularly challenging due to the typically hard lens nuclei. Thus, a comprehensive understanding of the underlying pathogenesis of HMC is imperative for the development of potential therapeutic methods [4].

Previous studies have reported that the highly methylated epigenetic modification of antioxidant and α-crystallin genes precipitates the early onset of HMC [3, 5]. Nonetheless, these studies have not fully elucidated the reason why HMC features severer nuclear opacity. Ferroptosis, a type of programmed cell death, first described in 2012 [6], is characterized by increased levels of redox-active iron and elevated lipid peroxidation. Recent research has shown its involvement in the formation of ARC [7–10]. Nonetheless, the role of ferroptosis in HMC remains unexplored. Given that HMC lens exhibits many features associated with ferroptosis, such as elevated malondialdehyde (MDA), increased oxidatively active substances, and reduced glutathione (GSH), compared with emmetropic eyes [11–13], it is plausible to consider ferroptosis as an essential mechanism underlying the development of HMC.

Notably, lenses from highly myopic eyes exhibit overgrowth and larger diameters than those from emmetropic eyes [14]. The Hippo pathway, a classical signaling pathway regulating organ size, becomes relevant in this context [15] because inhibiting the Hippo pathway leads to increased tissue/organ size [16, 17]. Moreover, discoidin domain receptor tyrosine kinase 2 (DDR2), which can be activated by extracellular matrix collagen, has been implicated in facilitating ferroptosis sensitivity through the Hippo pathway [18]. Based on this evidence, we hypothesize that DDR2 modulates ferroptosis through the Hippo pathway in HMC.

To corroborate our hypothesis, we assessed the ferroptosis sensitivity in HMC and conducted further mechanistic experiments. Our finding confirmed an elevated ferroptosis sensitivity in HMC, resulting from DDR2 overexpression. Additionally, we also demonstrated that DDR2 overexpression enhanced ferroptosis sensitivity mainly via the Src-Hippo pathway.

## Results

### Highly myopic lenses exhibit increased sensitivity to ferroptosis

**Fig. 1:**
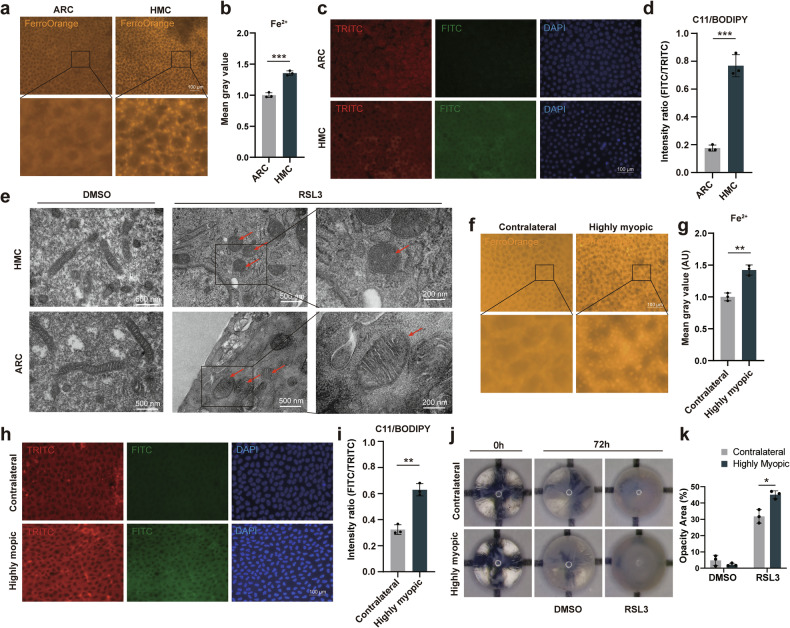
Highly myopic lenses are more sensitive to ferroptosis. **a**–**d** Evaluation of intrinsic Fe^2+^ and lipid peroxidation levels in lens epithelia from highly myopic cataract (HMC) and age-related cataract (ARC) patients (*n* = 3). For the FerroOrange, mean gray values were normalized to the average of the ARC group. For the C11/BODIPY, the ratio of gray values between the FITC and TRITC channels was calculated to represent the degree of lipid peroxidation. Scale bars: 100 μm. **e** Mitochondria observed using transmission electron microscopy in epithelia from HMC and ARC patients. Cells were treated with 1 μM RSL3 or DMSO for 4 h before being fixed. Red arrows show the mitochondria. Scale bars: 500 nm and 200 nm. **f**–**i** Assessment of Fe^2+^ (**f**, **g**) and lipid peroxidation (**h**–**i**) levels in lens epithelia of highly myopic and the contralateral eyes of mice (*n* = 3). Scale bars: 100 μm. **j**, **k** Representative photos and quantification of lens opacity from highly myopic and the contralateral eyes of mice before and after 10 μM RSL3 treatment. The opacity area (%) is defined as the ratio of the projection area of nuclear opacification to that of the whole lens. Data are presented as mean ± SD. Significance level was determined using unpaired *t*-tests (**b**, **d**) and paired *t*-tests (**g**, **i**, **k**). *P < 0.05, **P < 0.01, ***P < 0.001.

To test the hypothesis that lens epithelia from highly myopic eyes are more sensitive to ferroptosis, we assessed the levels of intrinsic Fe^2+^ and lipid peroxidation using FerroOrange and C11/BODIPY in human and mouse lens epithelia. In human lens epithelial samples, significantly elevated Fe^2+^ and lipid peroxidation levels were detected in HMC compared with ARC (Fig. 1a–d). Then, the Transmission Electron Microscopy (TEM) revealed that after a 4-hour treatment with 1 μM RSL3, an inducer of ferroptosis (dissolved in DMSO), lens epithelia from HMC exhibited more condensed mitochondria and deteriorated cristae (Fig. 1e), compared with the ARC group. To further validate this phenomenon, we constructed a lens-induced myopia mouse model, which also displayed heightened Fe^2+^ and lipid peroxidation levels in the highly myopic lens epithelia compared with the contralateral lens epithelia (Fig. 1f–i). Moreover, lens explants were cultured in vitro and after a 72-hour exposure to 10 μM RSL3, lenses from highly myopic eyes exhibited more severe nuclear opacity than the contralateral eyes (Fig. 1j, k). These findings support the notion of augmented ferroptosis sensitivity in highly myopic lenses.

### Upregulation of DDR2 enhances ferroptosis sensitivity in highly myopic lens

**Fig. 2:**
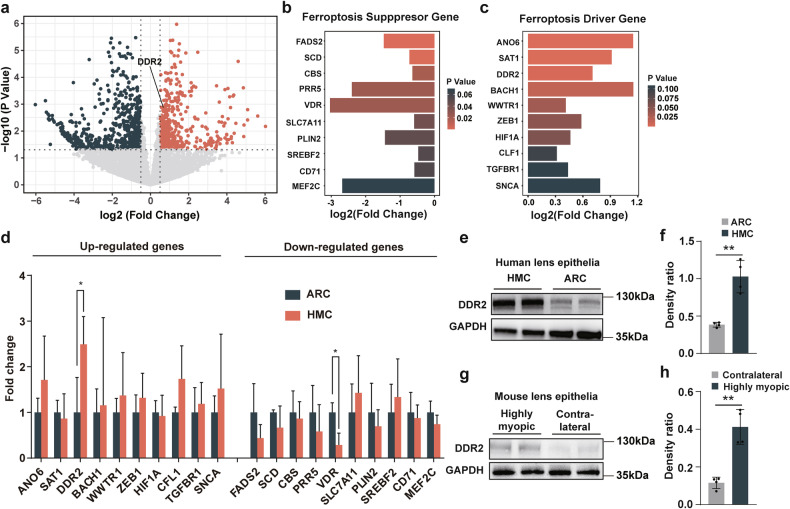
DDR2 is upregulated in highly myopic cataract as a ferroptosis-promoting gene. **a** The volcano plot showing significantly differentially expressed genes (log_2_[fold change] > 0.5 and p < 0.05) in lens epithelia from highly myopic cataract (HMC) and age-related cataract (ARC) patients. **b**, **c** Top ten driver genes showing upregulation (**b**) and top ten suppressor genes showing downregulation (**c**), arranged by *p*-values. **d** Quantitative PCR analysis of genes listed in b and c using lens epithelial samples from HMC and ARC patients (*n* = 3). **e**–**h** Western blotting detection of DDR2 protein levels in human (**e**, **f**) and mouse (**g**, **h**) lens epithelial samples (*n* = 4). Band densities were normalized to GAPDH for statistical analysis. Data are presented as mean ± SD. Multiple unpaired *t*-tests with False Discovery Rate post comparisons (**d**), unpaired *t*-tests (**f**), and paired *t*-tests (**h**) were used. *P < 0.05, **P < 0.01.

To investigate the mechanism underlying the heightened ferroptosis sensitivity of highly myopic lens, an RNA sequencing was conducted using human lens epithelial samples. After filtering out genes with low expression (Fig. S1a, b) and checking the mean-variance change (Fig. S1c), 479 significantly upregulated genes and 689 significantly downregulated genes were identified in HMC compared with ARC (Fig. 2a). Intersection of the significantly upregulated genes with known ferroptosis driver genes yielded 5 common genes (Fig. S1d), while intersection of significantly downregulated genes with suppressor genes yielded 8 common genes (Fig. S1e). Figure 2b, c illustrates the top ten driver genes showing upregulation and top ten suppressor genes showing downregulation in HMC, both selected based on the smallest p-values. Among these 20 genes, DDR2 and Vitamin D receptor (VDR) emerged as predominant genes (Fig. 2d), with only increased protein levels of DDR2 being verified subsequently (Fig. 2e–h). The expression level of VDR in lens epithelia is too low as revealed by quantitative PCR (qPCR). Besides, we found that cells treated with exogenous transforming growth factor β1 (TGF-β1) showed increased DDR2 expression (Fig. S2a, b) and enhanced ferroptosis sensitivity (Fig. S2c, d). TGF-β1 is a cytokine that has been proven to increase in the aqueous humor of highly myopic eyes [19], which may be a potential cause of DDR2 overexpression.

**Fig. 3:**
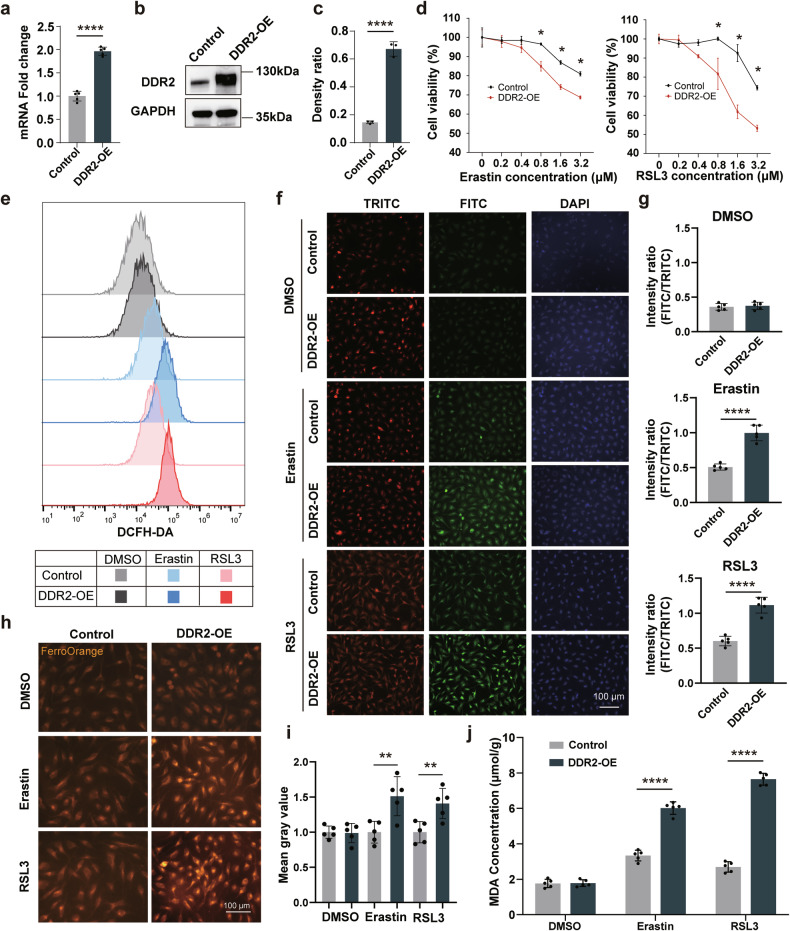
DDR2 enhances the ferroptosis sensitivity of lens epithelial cells. **a**–**c** Quantitative PCR (**a**) and Western blotting (**b**, **c**) analyses display the mRNA and protein levels of DDR2 after transduction using the DDR2 overexpressing lentivirus (qPCR, *n* = 5; Western blotting, *n* = 3). **d** Cell viability of DDR2-OE cells assessed by CCK-8 after 24 h treatment with varying concentrations of Erastin and RSL3 (n = 5). **e**–**j** Measurements of reactive oxygen species (**e**), lipid peroxidation (**f**, **g**), Fe^2+^ (**h**, **i**), and malondialdehyde (MDA) (**j**) levels using DCFH-DA probe, C11-BODIPY staining (*n* = 5), FerroOrange staining (*n* = 5) and an MDA assay kit (*n* = 5), respectively. Control and DDR2-OE cells were pretreated with 1 μM Erastin or RSL3 for 24 h. The quantification methods of FerroOrange and C11-BODIPY staining are as same as the description in Fig. 1. Scale bars: 100 μm. Band densities for Western blotting were normalized to the GAPDH for statistical analysis. Data are presented as mean ± SD. Multiple unpaired *t*-tests with False Discovery Rate post comparisons (**d**) and unpaired *t*-tests (**a**, **c**, **g**, **i**, **j**) were used. *P < 0.05, **P < 0.01, ****P < 0.0001.

Furthermore, to validate the impact of DDR2 on ferroptosis, DDR2 overexpressing (DDR2-OE) SRA 01/04 cell line (a human lens epithelial cell line) was constructed. Quantitative PCR (Fig. 3a) and Western blotting assays (Fig. 3b, c) confirmed the successful construction. CCK-8 indicated that DDR2-OE cells exhibited more declined cell viability (Fig. 3d) when treated with various concentrations of Erastin (another ferroptosis inducer) or RSL3, than the control group. Additionally, we also found elevated levels of Reactive oxygen species (ROS) (Fig. 3e), lipid peroxidation (Fig. 3f, g), Fe^2+^ (Fig. 3h, i), and MDA (Fig. 3j) in the DDR2-OE cells following treatment with Erastin or RSL3. Furthermore, Annexin-V/PI flow cytometry analysis (Fig. S3a) demonstrated that only Ferrostatin-1 (a ferroptosis inhibitor), but not Z-VAD-FMK (an apoptosis inhibitor) or Bafilomycin A1 (an autophagy inhibitor), significantly reduced the cell death induced by RSL3, suggesting that RSL3-induced cell death was attributed to ferroptosis rather than apoptosis or autophagy. Similarly, the caspase 3/7 staining (Fig. S3b) and CCK-8 assay (Fig. S3c, d) showed consistent results. Collectively, above findings demonstrate that DDR2 overexpression notably enhances ferroptosis susceptibility in highly myopic lens.

### DDR2 promotes ferroptosis sensitivity through the Hippo pathway

**Fig. 4:**
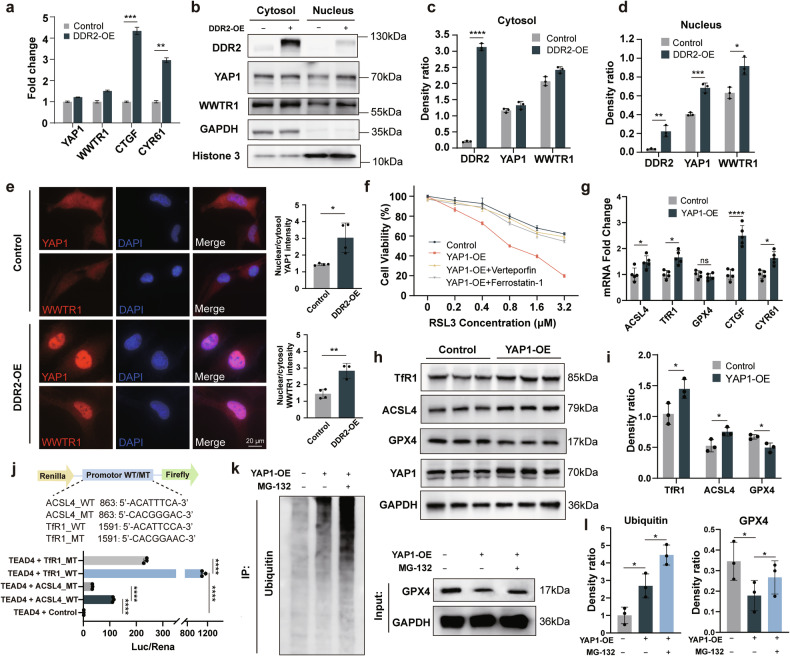
DDR2 overexpression promotes ferroptosis sensitivity through the Hippo pathway. **a** Quantitative PCR results of Hippo pathway related genes (YAP1, WWTR1, CTGF, and CYR61) in DDR2 overexpressing (DDR2-OE) cells (*n* = 3). **b**–**d** Western blotting and densitometry analysis of YAP1 and WWTR1 in the cytosol and nucleus of DDR2-OE cells (*n* = 3). **e** Immunofluorescence and nuclear/cytosol fluorescence intensity quantification of YAP1 and WWTR1 in cytosol and nucleus (*n* = 4). Scale bar: 20 μm. Each replicate was calculated from five randomly selected cells. **f** Cell viability measured by CCK8 (*n* = 3). Cells were treated with 2 μM verteporfin or 2 μM ferrostatin-1 along with various concentrations of RSL3 for 24 h. **g** Quantitative PCR results of ferroptosis-related genes (ACSL4, TfR1, and GPX4) and Hippo pathway downstream genes (CTGF and CYR61) in YAP1 overexpressing (YAP1-OE) cells (*n* = 5). **h**, **i** Western blotting results and densitometry analysis of ferroptosis-related genes of YAP1-OE cells. **j** Dual luciferase reporter assay results of transcription factor TEAD4 co-transfected with ACSL4 and TfR1 promotors (WT and MT refer to wild type and mutative type, respectively) (*n* = 3). **k** Immunoprecipitation result showing the increased GPX4 ubiquitination level and decreased GPX4 protein level in the YAP1-OE cells (*n* = 3). The decreased GPX4 protein level can be rescued by the treatment of 10 μM MG-132 for 6 h. **l** Densitometry analysis of k. Ubiquitin levels were normalized to the average value of the control group. Band densities of Western blotting assays were normalized to GAPDH for statistical analysis. Data are presented as mean ± SD. Statistical significance was determined using multiple unpaired *t*-tests with False Discovery Rate post comparisons (**a**, **c**, **d**, **g**, **i**), unpaired *t*-tests (**e**), and one-way ANOVA with the Dunnett’s multiple comparison test (j, **l**). *P < 0.05, **P < 0.01, ***P < 0.001, ****P < 0.0001.

A previous study has shown that DDR2 overexpression could regulate ferroptosis sensitivity through the Hippo pathway [18]. In light of this, we investigated the expression levels of two downstream effectors of the Hippo pathway: Yes-associated protein 1 (YAP1) and WW domain containing transcription regulator 1 (WWTR1, also known as TAZ), as well as two canonical target genes of YAP1/WWTR1: CTGF and CYR61. mRNA levels of CTGF and CYR61 were significantly elevated in DDR2-OE cells, while those of YAP1 and WWTR1 remained unchanged (Fig. 4a). Based on these initial findings, we delved deeper into the subcellular localization of YAP1 and WWTR1 by Western blotting (Fig. 4b, c) and immunofluorescence (Fig. 4e), revealing that DDR2 overexpression increased the nuclear levels of YAP1 and WWTR1.

To further elucidate how Hippo pathway regulates ferroptosis. We transfected cells with a plasmid carrying YAP1 gene with the S127A mutation, which specifically localizes YAP1 protein to the nucleus. YAP1 overexpression (YAP1-OE) cells exhibited enhanced YAP1 activity by increasing CTGF and CYR61 expression (Fig. S4a), and led to elevated ferroptosis sensitivity (Fig. 4f, Fig. S4b, c). This sensitivity could be rescued by Verteporfin (a YAP1 inhibitor) or Ferrostatin-1 (Fig. 4f). Then the protein and mRNA levels of three key ferroptosis-related genes: acyl-CoA synthetase long-chain family 4 (ACSL4), transferrin receptor protein 1 (TfR1), and glutathione peroxidase 4 (GPX4) were examined in YAP1-OE cells. We observed elevated ACSL4 and TfR1 protein levels and reduced GPX4 levels (Fig. 4h, i). Results of qPCR and dual-luciferase reporter assays confirmed that YAP1 cooperated with the transcription factor TEAD4 and directly binded to the promoters of ACSL4 and TfR1, enhancing their transcription activity (Fig. 4g, j). The unchanged GPX4 mRNA level suggested that YAP1 may have reduced GPX4 protein through post-translational modulation, as YAP1 has been shown to suppress the deubiquitinase USP31 [20]. Supporting this, immunoprecipitation assays revealed higher levels of ubiquitinated GPX4 in YAP1-OE cells, and treatment with the proteasome inhibitor MG132 restored GPX4 protein levels (Fig. 4k, l).

Moreover, similar gene expression changes were observed in primary lens epithelia transduced with DDR2-OE virus (Fig. S4d). Together, these results demonstrate that DDR2 enhances ferroptosis sensitivity via nuclear translocation of YAP1 and the subsequent regulation of ferroptosis-related genes.

### DDR2 regulates the Hippo pathway by interacting with Src

**Fig. 5:**
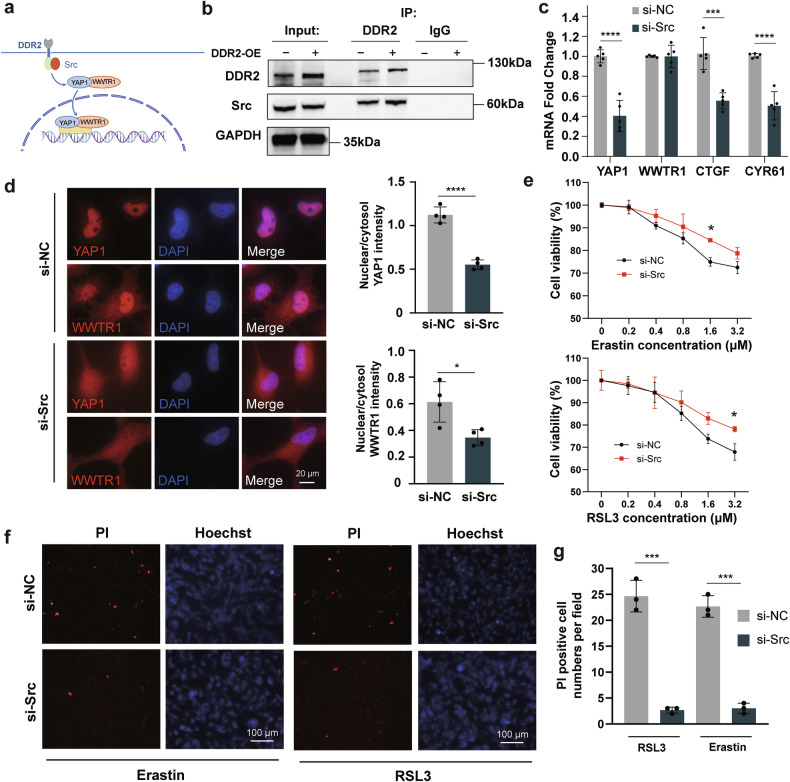
The Hippo pathway is modulated by DDR2 in a Src-dependent manner. **a** A schematic diagram of the DDR2-Src-Hippo pathway. **b** Co-immunoprecipitation result of the interaction between DDR2 and Src. The Src protein was pulled down by DDR2 antibody. **c** mRNA levels of Hippo-related genes after Src knockdown in DDR2 overexpressing (OE) cells evaluated by quantitative PCR (*n* = 5). **d** Immunofluorescence and nuclear/cytosol fluorescence intensity quantification of YAP1 and WWTR1 in DDR2-OE cells transfected with Src-targeted siRNA (*n* = 4). Each replicate was calculated from five randomly selected cells. Scale bar: 20 μm. **e** Cell viability evaluated by CCK-8. Cells were treated with varying concentrations of Erastin and RSL3 for 24 h (*n* = 3). **f**, **g** Typical Hoechst/PI staining photos of Src knockdown DDR2-OE cells and the control (*n* = 3). Cells were treated with 1 μM RSL3 for 24 h. Scale bar: 200 μm. Data are presented as mean ± SD. Multiple unpaired *t*-tests with False Discovery Rate post comparisons (**c**, **e**) and unpaired *t*-tests (**d**, **g**) were used. *P < 0.05, ***P < 0.001, ****P < 0.0001.

Then in order to elucidate how DDR2 regulated the Hippo pathway, we conducted a co-IP experiment to verify the interaction between DDR2 and a reported kinase Src [21]. Results revealed that Src could be pulled down by DDR2, with increased levels observed in DDR2-OE cells (Fig. 5b). A counter experiment using the Src antibody to pull down DDR2 yielded consistent results (Fig. S4f). Following this, we carried out a Src knockdown in DDR2-OE cells. Fig. S4g demonstrated the knockdown efficiency of three siRNAs and the siRNA-2 was adopted afterwards. Western blotting confirmed the reduced Src protein levels after knockdown (Fig. S4h). Notably, we also found that Src knockdown lowered the expression of YAP1, CTGF, and CYR61 (Fig. 5c), as well as diminished nuclear levels of YAP1 and WWTR1 (Fig. 5d), thereby rescued the heightened ferroptosis sensitivity in DDR2-OE cells (Fig. 5e–g). Collectively, these results indicate that DDR2 regulate the Hippo pathway by interacting with Src (Fig. 5a).

### Inhibiting DDR2 mediated ferroptosis mitigates the cataract formation in highly myopic mice

**Fig. 6:**
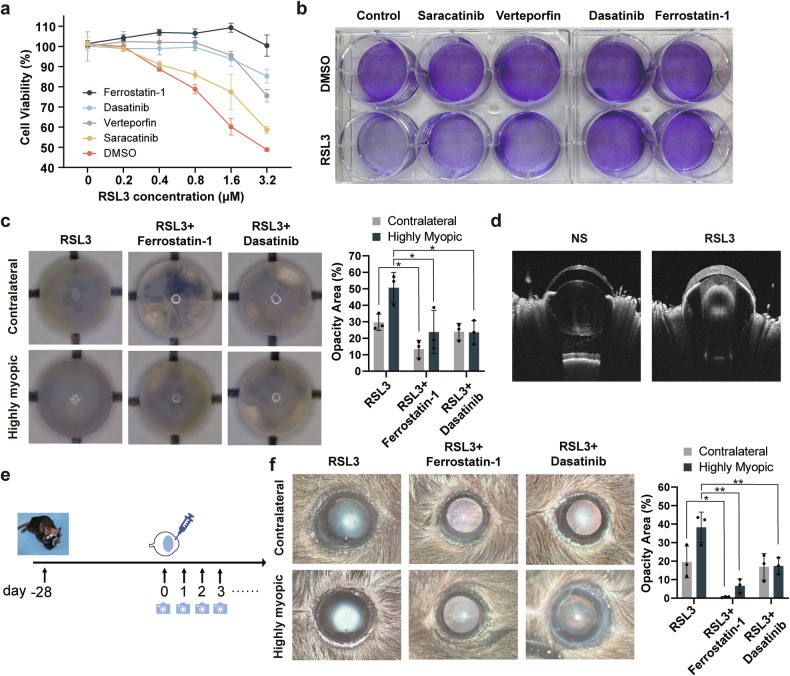
Inhibiting DDR2 mediated ferroptosis mitigates the highly myopic cataract (HMC) formation. **a**, **b** Cell viability of DDR2-OE cells measured by CCK-8 (**a**, *n* = 3) and crystal violet staining (**b**). Cells were exposed to RSL3 with or without Ferrostatin-1(2 μM), Dasatinib (2 μM), Verteporfin (2 μM), and Saracatinib (2 μM) for 24 h. **c** Typical pictures of lens explants after culturing in vitro for 72 h. Lenses were isolated from highly myopic and contralateral eyes of mice and cultured with 10 μM RSL3, with or without 10 μM Ferrostatin-1 or 10 μM Dasatinib (*n* = 3). The percentage of opacity area was quantified by the ratio of the projection area of nuclear opacification to that of the whole lens. **d** Optical coherence tomography pictures showing the typical nuclear opacity at the fifth day after being injected with 10 μM RSL3 (*n* = 3). **e** A schematic flow chart of mouse model construction of HMC. **f** Typical ocular pictures taken at the fifth day after anterior camber injection with 2 μL RSL3 (10 μM) with or without 10 μM Ferrostatin-1 or 10 μM Dasatinib in highly myopic and contralateral eyes of mice. The opacity area (%) was quantified by the ratio of the projection area of nuclear opacification to that of the cornea. Data are presented as mean ± SD. Statistical significance was determined using one-way ANOVA with the Dunnett’s multiple comparison test (**c**, **f**). *P < 0.05, **P < 0.01.

Experiments were conducted to investigate the potential of alleviating ferroptosis of lens epithelia through the DDR2-Src-Hippo pathway, and possible mitigation of HMC in a mouse model. As Fig. 6a showed, death of DDR2-OE cells induced by RSL3 could be rescued by Dasatinib (a DDR2 inhibitor), Verteporfin, Saracatinib (a Src inhibitor), and Ferrostatin-1 to varying extents. Crystal violet staining further confirmed these results (Fig. 6b). Lens explants extracted from highly myopic eyes and contralateral eyes were cultured with RSL3, with or without Ferrostatin-1 or Dasatinib. After 72 h, the highly myopic lens exhibited increased opacity, which could be mitigated by Ferrostatin-1 and Dasatinib (Fig. 6c). Furthermore, 10 μM RSL3 was injected to the anterior chamber to induce nuclear cataract formation in vivo. Fig. S5a illustrated a positive correlation between cataract severity and RSL3 concentration and exposure time. Also, on the fifth-day post-injection, the GSH/ Glutathione oxidized (GSSG) ratio in both lens cortex and nucleus significantly reduced (Fig. S5b), accompanied by obvious nuclear opacity as evaluated by optical coherence tomography (Fig. 6d). We further apply the RSL3 injection to the highly myopic model to mimic HMC in vivo, with flowchart depicted in Fig. 6e. Specifically, after wearing lenses for 28 days, spectacles were removed and 10 μM RSL3 were injected. Ocular photos taken at the fifth day after injection showed that highly myopic eyes developed more severe nuclear opacity than contralateral eyes in vitro, and the use of Ferrostatin-1 and Dasatinib could alleviate lens opacity (Fig. 6f). Together, these results show that inhibiting DDR2 activity and ferroptosis is able to alleviate ferroptosis-related HMC formation.

## Discussion

HMC is a leading cause of blindness, posing significant challenges to visual health. Yet, the precise pathological mechanisms underlying HMC remain elusive. Our study demonstrated that lens epithelia from highly myopic eyes were more sensitive to ferroptosis, which was a critical contributor to the severe nuclear opacity observed in HMC. Notably, we provided further evidence that the overexpression of DDR2 enhanced ferroptosis sensitivity, via the Src-Hippo signaling pathway. We also found that applying ferroptosis or DDR2 inhibitors can mitigate ferroptosis-associated HMC both in vivo and in vitro, offering promising therapeutic targets for potential HMC treatment.

Previous studies have highlighted that highly myopic eyes exhibit more characteristics related to ferroptosis compared with emmetropic eyes. For example, Micelli-Ferrari et al. reported lower GSH levels (essential for oxidative damage protection) in HMC compared to ARC [13]. Similarly, the subretinal fluid of highly myopic eyes contained significantly higher concentrations of lipid peroxidation products than emmetropic eyes [22]. Chen et al. identified activation of ferroptosis pathways in the myopic corneal stroma using proteomic analyses [23]. Consistent with these findings, our study confirmed that lens epithelial samples from HMC were more sensitive to ferroptosis. Notably, intraocular injection of RSL3, a ferroptosis inducer, successfully induced more severe nuclear cataract in highly myopic eyes compared to contralateral eyes. These results suggest that heightened ferroptosis sensitivity in lens epithelia contributes to the formation of severe lens nuclear opacity in highly myopic eyes.

We identified DDR2 as a key protein overexpressed in lens epithelia of HMC, contributing to increased ferroptosis sensitivity. DDR2-mediated signaling has been previously linked to the activation of Src kinase [24–27]. Lin et al. reported that DDR2 promoted ferroptosis sensitivity through the Src-Hippo axis in recurrent breast cancer [18]. Similarly, our findings revealed that DDR2 regulated the Hippo pathway in lens epithelia by activating Src. Specifically, the intracellular segment of DDR2 directly interacted with Src, leading to the nuclear translocation of YAP1 and WWTR1, two homologous transcriptional coactivators of the Hippo pathway.

The roles of YAP1/WWTR1 in regulating ferroptosis have been demonstrated in various studies, though their downstream targets differ. Yang et al. reported that WWTR1 knockdown reduced ferroptosis sensitivity through the regulation of ANGPTL4-NOX2 axis in ovarian cancer [28], and Wu et al. and He et al. reported that YAP1 promotes ferroptosis through the upregulation of ACSL4 [29, 30]. Additional studies link YAP1/WWTR1 to ferroptosis regulation via SKP2, ALOXE3, and TfR1 [31–33]. In this study, we observed that nuclear translocation of YAP1 enhanced ferroptosis sensitivity in lens by upregulating ACSL4 and TfR1, and downregulating GPX4. ACSL4, TfR1, and GPX4 are all important ferroptosis regulatory genes. ACSL4 activates long-chain polyunsaturated fatty acids and is essential for ferroptosis execution; TfR1, a membrane receptor component of transferrin, contributes to the cellular iron pool required for ferroptosis; GPX4 serves to interrupt lipid peroxidation. More specifically, the nuclear translocation of the YAP1 protein promoted the transcription of ACSL4 and TfR1 by binding directly to their promoters with TEAD4 and reduced the GPX4 protein level post-translationally by increasing its ubiquitination.

Interestingly, we observed that lens epithelia treated with exogenous TGF-β1 increased DDR2 expression and ferroptosis sensitivity, in line with previous studies [34, 35]. As reported earlier, TGF-β1 levels in the aqueous humor of highly myopic eyes are higher than those in emmetropic eyes [14, 19, 36]. We speculate that this may be one of the potential reasons of increased DDR2 expression in lens epithelia of highly myopic eyes.

**Fig. 7:**
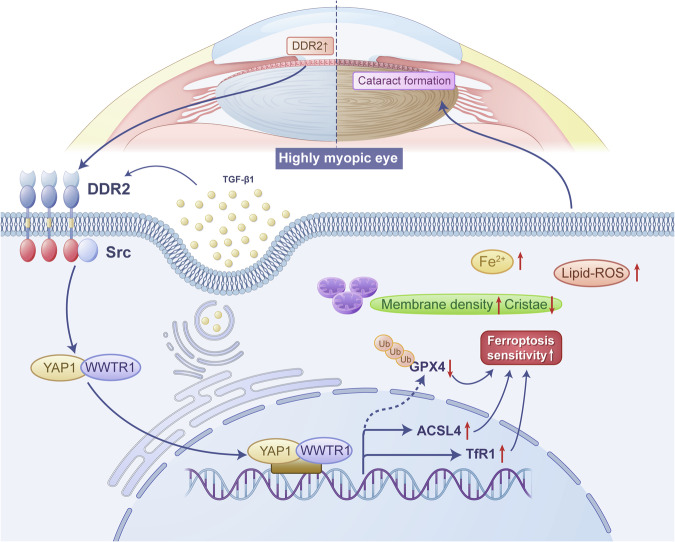
Schematic plot of DDR2 enhancing ferroptosis sensitivity via the Src-Hippo pathway in highly myopic cataract. In highly myopic eyes, the elevated levels of TGF-β1 in the lens microenvironment contribute to an increased expression of DDR2. This, in turn, activates the Src kinase, which promotes the nuclear translocation of key effectors of the Hippo pathway: YAP1 and its homolog WWTR1. YAP1 upregulates the transcription of ACSL4 and TfR1, while simultaneously downregulating GPX4 at the post-translational level, which collectively enhance the lens’s sensitivity to ferroptosis, manifesting as increased levels of Fe^2+^, lipid peroxidation, and mitochondrial distortion. Ultimately, the heightened ferroptosis sensitivity accelerates the formation of nuclear cataracts in highly myopic lenses.

In conclusion, our study unveiled the functional and mechanistic role of ferroptosis in HMC pathogenesis. We identified that DDR2 overexpression in highly myopic eyes contributed to the enhanced ferroptosis sensitivity via the Src-Hippo pathway (Fig. 7). In vivo anterior chamber injection of RSL3 induced more severe lens nuclear opacity in a highly myopic mouse model, which could be partially ameliorated by ferroptosis and DDR2 inhibitors. These findings underscore DDR2-mediated ferroptosis as a potential therapeutic target for HMC intervention.

## Materials and methods

### Human lens capsule samples

In this study, high myopia was identified as eyes with axial length of ≥26.00 mm, while emmetropia was defined as eyes with axial length ranging from 22.00 mm to 24.50 mm. During standard cataract surgeries, anterior lens capsules were peeled off during capsulorhexis. Specimens were immediately spread on culture dishes for cell staining, or treated with ferroptosis inducers, or stored at −80 °C until further experiments (RNA extraction and protein isolation). For qPCR, a single piece of lens capsule constituted one sample. For Western blotting, 3 pieces of lens capsules were pooled to provide sufficient protein for robust analysis.

### TEM

For TEM analysis, lens capsules obtained from surgeries were immediately spread on the filter paper and then treated with 1 μM RSL3 or DMSO for 4 h. The samples were then fixed overnight in a combination of 2% paraformaldehyde and 2.5% glutaraldehyde. Then steps including washing, dehydration, and embedding in resin, were performed according to established protocols. Embedded samples were sectioned using a Leica EM UC7 ultramicrotome (Leica, Wetzlar, Germany). The morphology of mitochondria was examined under a Hitachi HT7800 electron microscope (Tokyo, Japan) operating at 80 kV.

### Lens-induced high myopia mouse model

Four-week-old male C57BL/6 mice were bought from Chengxi Biotech Company (Shanghai, China), reared with free access to water and food under a 12-h light-dark cycle. In all procedures, the mice were anesthetized using a solution of 1% sodium pentobarbital (100 mg/kg, i.p.; Sanshu, Beijing, China) and 10 mg/kg xylazine (Huamu, Beijing, China). An infrared photorefractor (Striatech, Germany) was used to assess the refractive state. Only mice displaying less than 1.00 D of refractive disparity between two eyes were selected for inclusion. Theses mice were randomly allocated to the high myopia group or the control group using random number tables. As we previously reported [37, 38], defocus-induced high myopia model was established by affixing spectacles to the skull of the mouse. The right eye was fitted with a − 30 D lens, while the left eye wore a 0 D lens as a control. Daily checks ensured the spectacles remained securely attached. After 4 weeks, the refractive states of the mice were measured again. Mice showing a myopic shift of at least 6.00 D in the right eye compared to the left eye were considered successful models of defocus-induced high myopia. Due to visibly distinguishable treatments, blinding was not used. Then lens capsules were harvested for fluorescence staining or Western blotting. In Western blotting, 4 pieces of capsules were pooled to provide sufficient protein for robust analysis.

### Lens explant

Eyes from successful high myopia models were harvested, disinfected with iodophor and immediately submerged in normal saline maintained at 37 °C. Then the lenses were extracted and cultured in high glucose Dulbecco’s Modified Eagle Medium (DMEM; 11965092; Gibco, Carlsbad, CA, USA) medium containing 20% Fetal Bovine Serum (SV30208; Cytiva, Marlborough, MA, USA), 100 units/mL penicillin and 100 µg/mL streptomycin (both 15140122; Gibco) under the condition of 5% CO_2_ at 37 °C. Lenses were exposed to reagents according to the experiment design and images were captured using a stereomicroscope against a black cross background. Referring to the approach proposed by Kubo et al and Takashima et al. [39, 40], nuclear cataract extent was quantified by calculating the ratio of the projection area of nuclear opacification to that of the whole lens using the ImageJ software.

### Gene expression analysis by RNA sequencing

Total RNA was extracted using the TRIzol reagent (Invitrogen, CA, USA) according to the manufacturer’s protocol. RNA purity and quantification were evaluated with the NanoDrop ND-1000 spectrophotometer (Thermo Scientific, USA). RNA integrity was assessed using the Agilent 2100 Bioanalyzer (Agilent Technologies, Santa Clara, CA, USA). Libraries were then constructed using the TruSeq Stranded Total RNA Library Prep Kit (Illumina, USA) following the manufacturer’s instructions. Sequencing was performed on an Illumina Novaseq 6000 platform, generating 150 bp paired-end reads. Raw reads in fastq format were firstly processed using fastp [41], and low-quality reads were removed to yield clean reads. These clean reads were mapped to the reference genome using HISAT2 [42]. Differential gene expression was analyzed using the limma statistical package (version 3.26.9), as described at Bioconductor (http://www.bioconductor.org). Differentially expressed genes were defined by an absolute log_2_(fold change) value > 0.5 and a *p-*value < 0.05. These genes were then compared with ferroptosis driver and suppressor genes from FerrDb website to identify intersectant genes [43].

### Cell culture

SRA 01/04 cells, sourced from Shanghai Jinyuan Biotechnology, were authenticated using STR DNA profiling. Cells were cultured in high glucose DMEM (11965092; Gibco) supplemented with 10% FBS (SV30208; Cytiva) and 1% antibiotics (15140122; Gibco) in a humidified incubator at 37 °C and 5% CO_2_. Routine PCR testing for mycoplasma ensured the purity of the cultures.

To explore the influence of DDR2 on ferroptosis, we established DDR2-OE, YAP1-OE, and Src knockdown SRA 01/04 cell lines. DDR2-OE cells were established by transducing a lentivirus carrying a DDR2-OE plasmid obtained from Genechem (Shanghai, China). Cells were selectively cultured with 2 μg/mL puromycin (ST551; Beyotime, Shanghai, China) to achieve stable transduction.

The YAP1 overexpressing cells were obtained by transiently transfected YAP1 with S127A mutation plasmids purchased from Genechem (Shanghai, China). The primers used for the acquisition of the target gene fragment are: forward primer (5’ to 3’: CACACTGGACTAGTGGATCCCGCCACCATGGATCCCGGGCAG) and reverse primer (5’ to 3’: AGTCACTTAAGCTTGGTACCGATAACCATGTAAGAAAGCTTTCTTTATC). Lipofectamine 3000 (L3000008; Thermo Scientific) and Opti-MEM (31985070, Gibco) were used in the transfection. After transfection, in some cases, MG-132 (HY-13259; MCE, Shanghai, China) was added to inhibit proteosome function.

The Src knockdown cells were obtained using Src targeted siRNAs purchased from Ribio Company (Guangzhou, China). The sequences of siRNA-1 to siRNA-3 are GTTGTATGCTGTGGTTTCA, CTCGGCTCATTGAAGACAA and GAGAGAACCTGGTGTGCAA, respectively. For subsequent analysis, cells were harvested for RNA extraction after 24 h and for protein extraction after 48 h.

### Cell viability

Cell viability was assessed using the CCK-8 (C0037; Beyotime, China). Cells were seeded in 96-well plates (5000 cells/well) for 24 h. The medium was then replaced with Erastin (HY-15763; MCE, Shanghai, China), RSL3 (HY-100218A; MCE), or inhibitors including Ferrostatin-1 (HY-100579; MCE), Dasatinib (HY-10181; MCE), Saracatinib (HY-10234; MCE), Verteporfin (HY-B0146; MCE) at specific concentrations. After 10 µL of CCK-8 was added to each well for an hour, absorbance at 450 nm was measured using a spectrophotometer (Tecan Spark, Tecan, Shanghai, China). Cells without treatment served as the negative control, while solutions containing DMEM and CCK-8 without cells were used as blank control. Cell viability (%) = (A_450, sample_ − A_450, blank_)/ (A_450, control_ − A_450, blank_) × 100%.

Hoechst 33342/PI (HO/PI) staining was performed using a staining kit (C1056; Beyotime). Cells were cultured on confocal dishes before incubated with 5 µg/ml HO and 5 µg/ml PI at 37 °C, 5% CO_2_ for 15 min. Afterward, cells were washed and examined under a fluorescence microscopy.

Apoptotic cell death was analyzed by Annexin V/Propidium Iodide (PI) double staining kit (BL110A; Biosharp) by flow cytometry. Annexin V( − )PI(−), annexin V( + )PI(−), annexin V( + )PI(+), and annexin V( − )PI(+) cells were defined as viable, early apoptotic, late apoptotic, and necrotic cells, respectively. Intracellular caspase 3/7 was also detected to assess apoptosis using a living cell detection agent (C10432; Thermofisher).

### Intracellular iron and lipid peroxidation

Intracellular ferrous iron was stained using a 1 μM solution of FerroOrange (F374; Dojindo, Japan). Capsules obtained from surgery were immediately spread on the confocal dish. After gently rinsing away the viscoelastic substance and being exposed to corresponding treatments, they were covered with the working solution of FerroOrange and incubated for 30 min at 37 °C. Fluorography was then captured. A similar procedure was applied to the SRA 01/04 cells. Each image was captured using the same exposure time.

For lipid peroxidation analysis, C11-BODIPY 581/591 (HY-D1301; MCE) staining was conducted. Briefly, a 10 mM solution of C11-BODIPY was prepared in DMSO and then diluted in HBSS to achieve a final concentration of 10 μM. Cells or capsules were incubated in the working solution for 30 min at 37 °C. Fluorescence photos were captured under the emission wavelength of 525 nm and 624 nm.

Quantitative data were analyzed using ImageJ (version 1.53k; National Institutes of Health, Bethesda, MD, USA). Images were first split into color channels, and only channels of interest were processed further. Then a threshold was applied to exclude background signal. Subsequently, the mean gray value within the threshold area was calculated using the “Measure” function. Each replicate came from the average value of three regions of interest that were randomly selected. For Fe^2+^ analysis, to minimize batch effects between replicates, gray values from each replicate were normalized to the average gray value of the ARC group. For lipid peroxidation analysis, the ratio of gray values between the FITC and TRITC channels was calculated to represent the degree of peroxidation.

### ROS, MDA and GSH/ GSSG

The 2’7’-dichlorodihydrofluorescein diacetate (DCFH-DA) kit (S0033S, Beyotime, Shanghai, China) was used to assess intracellular ROS levels. After treatment, cells were stained with 5 μM of DCFH-DA at 37 °C for 30 min. Excess probe was then washed out and labeled cells were trypsinized and analyzed by a flow cytometry (MoFlo XDP; Beckman Coulter, USA). Results were analyzed using the Flowjo software (version 10.8.1; https://www.flowjo.com/solutions/flowjo).

MDA levels were measured using an MDA Content Test Kit (S0131S; Beyotime) following the manufacturer’s instruction. The absorbance of each sample was measured at 532 nm, with the 450 nm absorbance serving as a reference. MDA concentration was normalized to the total protein content, determined using the bicinchoninic acid (BCA) Protein Assay Kit (P0012; Beyotime).

The GSH/GSSG ratio of lens cortex and nucleus was measured using a commercial kit (S0053; Beyotime) according to the manufacturer’s protocol. The GSH or GSSG concentration was normalized to the wet weight of the tissue.

### qPCR

Total RNA from the lens epithelial samples was extracted using the Trizol reagent (15596018CN; Thermo Fisher Scientific), while that from cell lines was extracted using an RNA purification kit (EZB-RN4; EZBioscience, Roseville, CA, USA). RNA quantitation and quality assessment were performed using a Nanodrop spectrophotometer (Thermo Fisher Scientific). Subsequently, RNA was reverse transcribed into cDNA using the HiFiScript gDNA Removal RT Master Mix (CW2020M, Cowin Biosciences, Shanghai, China). The mRNA levels of selected genes were quantified by SYBR Green-based qPCR on a BioRad CFX96 real-time PCR machine. The primer sequences used in this study are listed in the Supplemental Table 1.

### Western blotting

Western blotting analysis was performed as previously described [14]. Briefly, cells or tissues were collected and lysed in RIPA buffer containing 2% phosphatase and protease inhibitors (P1045, Beyotime, Shanghai, China). The capsule tissues were ground at −20 °C for 5 min to make protein fully released. Lysates were centrifuged at 12000 × *g* for 15 min, and the supernatants were collected. The Nucleoprotein Extraction Kit (C500009, Sangon, Shanghai, China) was used to isolate the nucleic protein. Protein concentrations were determined using the BCA Protein Assay Kit as mentioned before. Proteins were separated by 4–12% SDS-PAGE and subsequently transferred to PVDF membranes. The membranes were then blocked with 5% skimmed milk and incubated with primary antibodies under 4 °C overnight, followed by a 1 h incubation with horseradish peroxidase-conjugated secondary antibodies the next day. Signal visualization and band intensity were assessed using chemiluminescence on an imaging system (Bio-Rad, Hercules, CA, USA). The primary antibodies and dilution factors used are listed in Supplementary Table S2.

### Co-IP and IP

Co-IP was conducted according to the protocol provided with the Immunoprecipitation Kit (P2179S, Beyotime). Briefly, cells were lysed using the cell lysis buffer and centrifuged at 12,000 × *g* for 5 min at 4 °C. The supernatant was divided into two parts: one served as the positive control (input), another was first incubated with magnetic beads combined with normal control IgG to reduce nonspecific binding. And then the supernatants were incubated with magnetic bead conjugated with targeting antibodies. Subsequently, the beads were separated using a magnetic stand and washed with chilled lysis buffer for five times. To elute the antigen-antibody complex, beads were heated at 99 °C in 1× loading buffer for 5 min. The supernatant after magnetic separation was then analyzed by Western blotting.

### Dual-luciferase reporter assay

Putative TEAD4 binding regions in the ACSL4 and TfR1 promoters were identified using the JASPAR database (https://jaspar.elixir.no/#). Target fragments were amplified via PCR and inserted into the pGL3-basic luciferase reporter vector (GM-4629, Genomeditech, Shanghai, China) using seamless cloning. HEK-293 cells were co-transfected with TEAD4 plasmids and luciferase reporter plasmids (wild type [WT] and mutative type [MT]) using Lipofectamine 2000 (ThermoFisher). After 48 h, cell lysates were collected and assayed using a Dual-Luciferase Assay kit (GM-040502A, Genomeditech), following the manufacturer’s instruction. Luciferase activity was expressed as the ratio of firefly luciferase to renilla luciferase and normalized to the control group.

### Immunofluorescence

Immunofluorescence was performed as described previously [38]. Briefly, cells seeded on a confocal dish were fixed in 4% paraformaldehyde, permeabilized with PBS containing 0.5% Triton X-100, and blocked with 3% bovine serum albumin. Then cells were then probed overnight at 4 °C with antibodies against YAP1 and WWTR1, followed by incubation with a secondary antibody at room temperature for 1 h. Fluorescence pictures were captured using a confocal microscopy (TCS SP5; Leica Microsystems, Germany). Fluorescence intensity of cytosol and nucleus was measured using ImageJ. Specifically, the image was split into three channels (red, green, and blue). In the red channel, the entire cell region was selected using the “Threshold” function and saved to the “Region of Interest” (ROI) manager. The nuclear region was identified in the DAPI (blue) channel and similarly added to the ROI manager. Then in the red channel, cytoplasmic fluorescence intensity was calculated by subtracting the nuclear fluorescence intensity from the total cell fluorescence intensity. The nuclear-to-cytosol intensity ratio was subsequently determined.

### Cataract model using RSL3

We used anterior chamber injection of RSL3 to mimic the ferroptosis-related cataract formation. The anterior chamber injection protocol was performed as described before [44]. Briefly, after surface anesthesia and pupil dilation, the cornea is punctured closely anterior to the iridocorneal angle using a 30-gauge needle. Then a 1 μL air bubble is created by a 34-gauge blunt needle fitted in a 5 μL micro syringe (Hamilton, Reno, NV, USA) to seal the puncture site. Subsequently, reagents were injected according to the study design. The air bubble would be absorbed within 24 h. During the following days, optical photos after pupil dilation were taken under an operating microscope to record the opacity of lens. The degree of cataract was quantified by calculating the ratio of the projected area of the nuclear opacity to the total projected area of cornea using the ImageJ software. The optical coherence tomography (OCT) pictures were taken at the fifth day since injection using a swept-source OCT based biometer (YG-100 K, TowardPi Medical Technology Ltd, China).

### Statistical analysis

Results are expressed as mean ± SD. Statistical analyses were conducted using GraphPad Prism software (version 8). The normality was assessed with the Shapiro-Wilk test. The variance between the groups was assessed by the F test. Data from two groups were compared using two-tailed unpaired *t*-tests for unpaired experiments, or paired *t*-tests for self-control mouse experiments. The False Discovery Rate method is used in multiple comparisons to control the number of false positives. Data from three or more groups were compared using one-way ANOVA with the Dunnett’s multiple comparison test. A *P*-value < 0.05 was considered statistically significant. All results have been validated by three or more independent experiments based on the information gathered from assays conducted previously by our group. Details of number of replicates are provided in the individual figure legends.

## Supplementary information

Supplementary figures and legends

Supplemental tables

Original data file

## Data Availability

The mRNA sequencing data used in this study have been deposited in the Gene Expression Omnibus (GEO) under the accession code GSE244037. Original data can be found in the “Supplemental Material” part which is available at Cell Death & Disease’s website.
