# Supplementary material for: Enhanced ferroptosis sensitivity promotes the formation of highly myopic cataract via the DDR2-Hippo pathway
Source: Cell Death Dis. 2025 Feb 3;16(1):64. doi: 10.1038/s41419-025-07384-8 (PMC11790942; doi:10.1038/s41419-025-07384-8)
Supplement: Supplementary file 1 — Supplementary figures and legends [file 41419_2025_7384_MOESM1_ESM.docx]

**Supplementary figures**

**
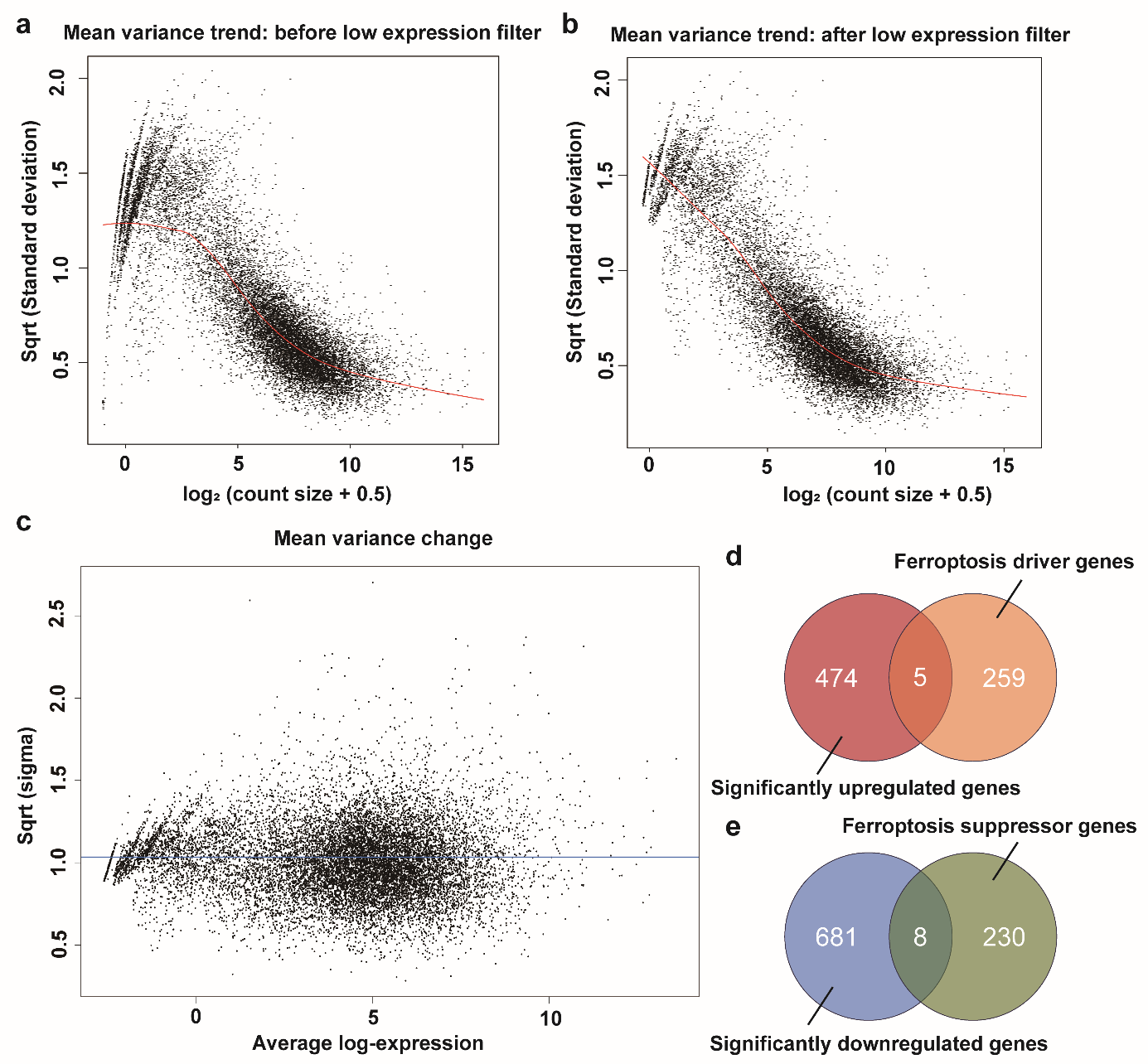
Fig. S1**

**Fig. S1 Processing and screening of the RNA sequencing data. a-b** Mean variance trend check before (a) and after (b) the low expression filtration. **c** Stability of mean variance change among different expression levels. **d** Intersection between significantly upregulated genes and ferroptosis driver genes. **e** Intersection between significantly downregulated genes and ferroptosis suppressor genes.

**
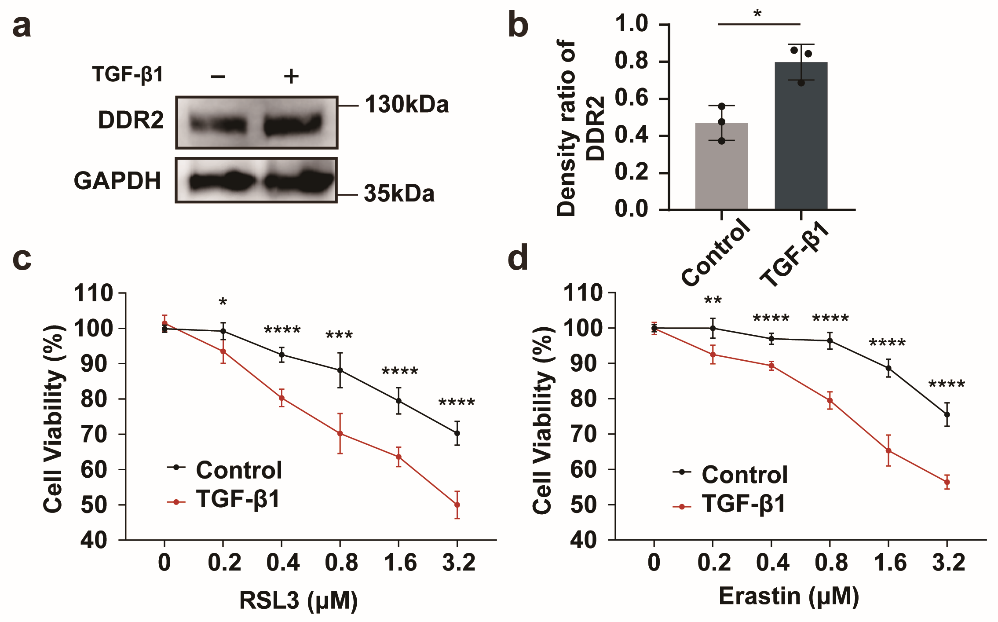
Fig. S2**

**Fig. S2 TGF-β1 treatment increased DDR2 expression and ferroptosis sensitivity. a-b** The DDR2 protein level of SRA 01/04 cells evaluated by Western blotting with and without a pretreatment of 20 ng/mL TGF-β1 for 72 h. **c-d** Cell sensitivity to 24 h treatment of varying concentrations of RSL3 and Erastin after treatment with TGF-β1 as described in d-e. Western blotting was normalized to the GAPDH for statistical analysis. Data are presented as mean ± SD. Statistical significances were determined using multiple t-tests with False Discovery Rate post comparisons (c-d) or unpaired t-tests (b). *P < 0.05, **P < 0.01, ***P < 0.001, ****P < 0.0001.

**
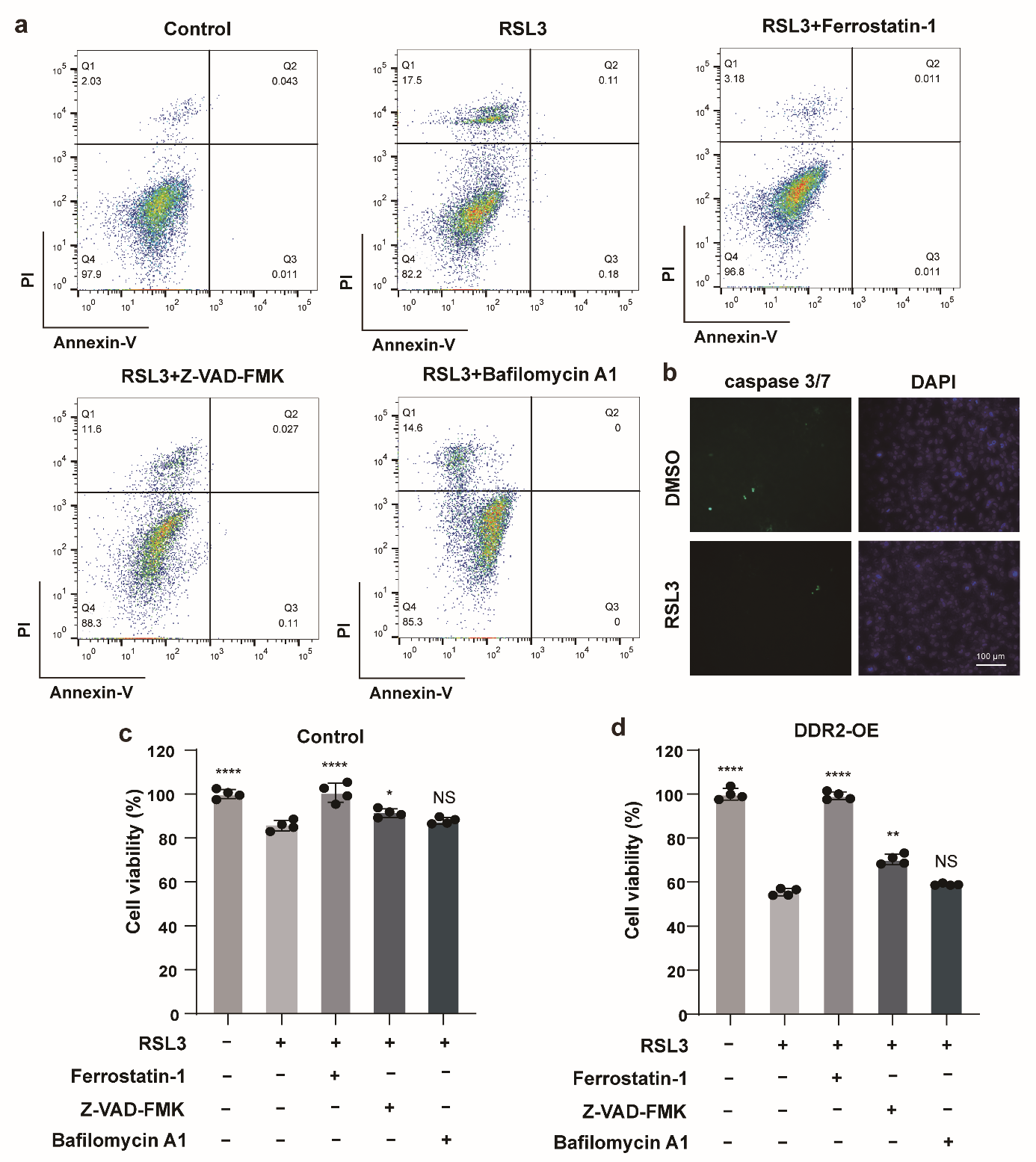
Fig. S3**

**Fig.S3 RSL3 induced ferroptosis rather than apoptosis or autophagy. a** Representative results from Annexin-V/PI staining evaluated by flow cytometry. SRA 01/04 cells were pretreated with 1 μM RSL3 (except the Control group) with or without 2 μM Ferrostatin-1, 2 μM Z-VAD-FMK and 2 μM Bafilomycin A1 for 24 h. **b** Living cell caspase 3/7 staining of cells treated with RSL3 (1 μM for 24 h) or equal amounts of DMSO. Scale bar: 100 μm. **c-d** Cell viability assessed by CCK-8 of DDR2 overexpressing and control cells (n = 4, statistical significance compared to the group treated with only RSL3). Cell pretreatments were the same as the description in a. Statistical significance was determined using the one-way ANOVA with the Dunnett’s multiple comparison test (c-d). *P < 0.05, **P < 0.01, **** P < 0.0001.


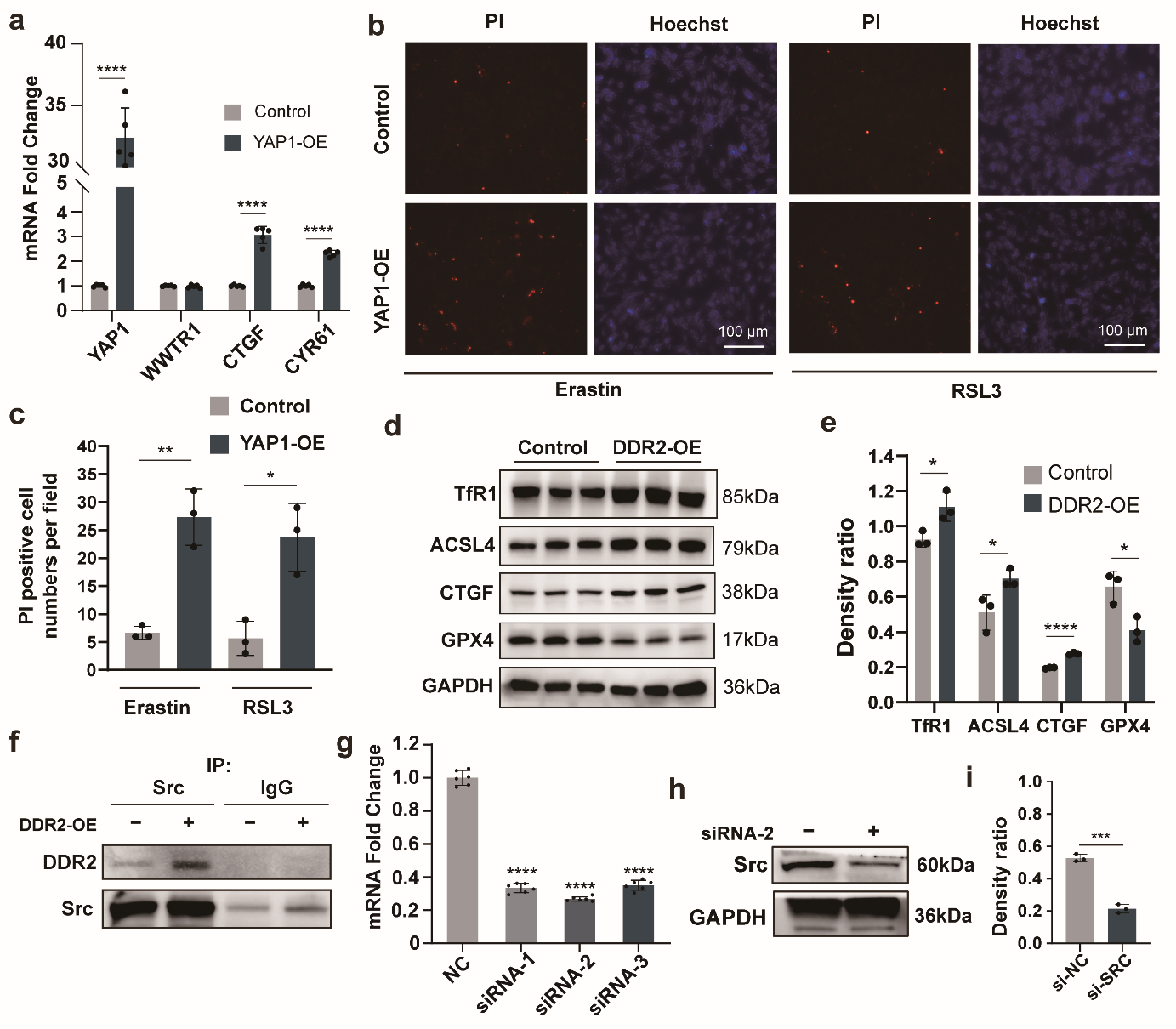
**Fig. S4**

**Fig. S4 DDR2 enhances ferroptosis via the Src-Hippo pathway. a** Quantitative PCR results of Hippo pathway related genes in cells transfected with YAP1 S127A plasmids (n = 5). **b-c** Typical HO/PI staining pictures of YAP1 overexpressing and the control (n = 3). Cells were pretreated with 1 μM Erastin or RSL3 for 24 h. Scale bar: 100 μm. **d-e** Western blotting results and densitometry analysis of ferroptosis related genes in DDR2 overexpressing primary human lens epithelia (n = 3). **f** Co-immunoprecipitation results of the interaction between DDR2 and Src (n = 3). The DDR2 protein was pulled down by Src antibody. **g** Knockdown efficiency of three Src targeted siRNA measured by quantitative PCR assays (n = 6). **h-i** Western blotting detection of Src protein after transfected with the siRNA-2 in h (n = 3). Western blotting was normalized to the GAPDH for statistical analysis. Data are presented as mean ± SD. Statistical significance was determined using unpaired t-tests with False Discovery Rate post comparisons (a,e), unpaired t-tests (c,i), and one-way ANOVA with the Dunnett’s multiple comparison test (g) and. *P < 0.05, **P < 0.01, ***P < 0.001, **** P < 0.0001.

**
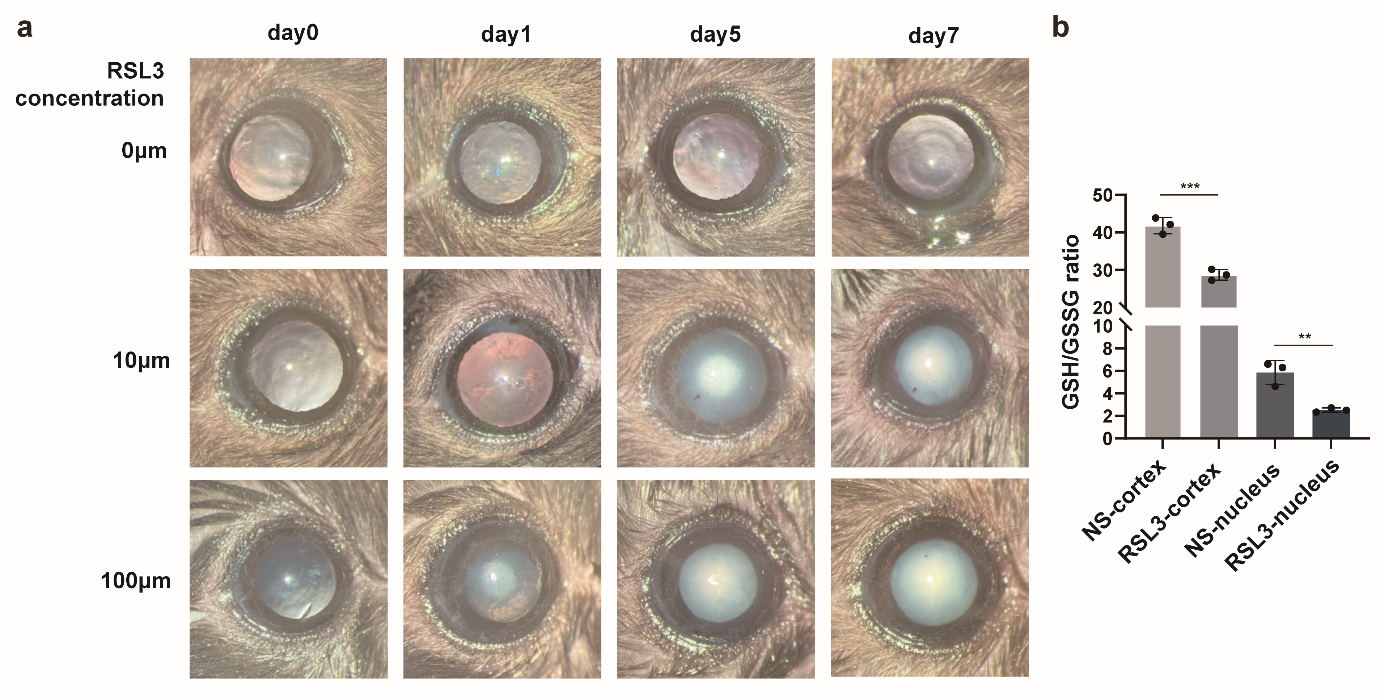
Fig. S5**

**Fig. S5 Model construction and evaluation of RSL3 induced nuclear cataract. a** Ocular pictures showing the formation of cataract. Photos were taken at initial and the first, fifth and seventh day after the anterior chamber injection of different concentration of RSL3. **b** GSH/GSSG ratios of the cortex and nucleus of lenses obtained at the fifth day from eyes injected with normal saline or 10 μM RSL3 (n = 3). Statistical significance was determined using unpaired t-tests (b). **P < 0.01, ***P < 0.001.
