# Supplementary material for: Enhanced ferroptosis sensitivity promotes the formation of highly myopic cataract via the DDR2-Hippo pathway
Source: Cell Death Dis. 2025 Feb 3;16(1):64. doi: 10.1038/s41419-025-07384-8 (PMC11790942; doi:10.1038/s41419-025-07384-8)
Supplement: Supplementary file 2 — Supplemental tables [file 41419_2025_7384_MOESM2_ESM.docx]

**Supplementary Tables**

**Table S1 Primer sequences for qRT-PCR**

| **Name** | **Forward (5’ – 3’)** | **Reverse (5’ – 3’)** |
| --- | --- | --- |
| ACSL4 | TCTAGTGAAGTTACAAGCAGGAGA | TCACATAGGACTGATCACTTTTGG |
| ANO6 | TGTCCCCGATTTGGGATCACT | CGTATGCTTGTCTTTTCCTCCT |
| BACH1 | CTCAGCCTTAATGACCAGCGG | GCCTACGATTCTTGAGTGGAAG |
| CBS | GGGGCTGAGATTGTGAGGAC | CGGTACTGGTCTAGGATGTGA |
| CD71 | TCGTGAGGCTGGATCTCAAAA | CCTTACTATACGCCACATAACCC |
| CFL1 | TTCAACGACATGAAGGTGCGT | TCCTCCAGGATGATGTTCTTCT |
| CTGF | CAGCATGGACGTTCGTCTG | AACCACGGTTTGGTCCTTGG |
| CYR61 | TGGTCAAAGTTACCGGGCAG | GGCTCCATTCCAAAAACAGGG |
| DDR2 | CCAGTCAGTGGTCAGAGTCCA | GGGTCCCCACCAGAGTGATAA |
| FADS2 | TGACCGCAAGGTTTACAACAT | AGGCATCCGTTGCATCTTCTC |
| GPX4 | GAGGCAAGACCGAAGTAAACTAC | CCGAACTGGTTACACGGGAA |
| HIF1A | ATCCATGTGACCATGAGGAAATG | TCGGCTAGTTAGGGTACACTTC |
| MEF2C | CCAACTTCGAGATGCCAGTCT | GTCGATGTGTTACACCAGGAG |
| PLIN2 | ATGGCATCCGTTGCAGTTGAT | GGACATGAGGTCATACGTGGAG |
| PRR5 | CCTTCACCCATTCCTGCATCC | AGAGGCGTGTTGTAGCTCTTG |
| SAT1 | ACCCGTGGATTGGCAAGTTAT | TGCAACCTGGCTTAGATTCTTC |
| SCD | TTCCTACCTGCAAGTTCTACACC | CCGAGCTTTGTAAGAGCGGT |
| SLC7A11 | TCTCCAAAGGAGGTTACCTGC | AGACTCCCCTCAGTAAAGTGAC |
| SNCA | AAGAGGGTGTTCTCTATGTAGGC | GCTCCTCCAACATTTGTCACTT |
| SRC | GAGCGGCTCCAGATTGTCAA | CTGGGGATGTAGCCTGTCTGT |
| SREBF2 | CCTGGGAGACATCGACGAGAT | TGAATGACCGTTGCACTGAAG |
| TGFBR1 | ACGGCGTTACAGTGTTTCTG | GCACATACAAACGGCCTATCTC |
| VDR | GTGGACATCGGCATGATGAAG | GGTCGTAGGTCTTATGGTGGG |
| WWTR1 | TCCCAGCCAAATCTCGTGATG | AGCGCATTGGGCATACTCAT |
| YAP1 | TAGCCCTGCGTAGCCAGTTA | TCATGCTTAGTCCACTGTCTGT |
| ZEB1 | TTACACCTTTGCATACAGAACCC | TTTACGATTACACCCAGACTGC |

qRT-PCR: Quantitative reverse transcription PCR.

**Table S2 Information for the primary antibodies used in this study.**

| **Assay** | **Antibody** | **Company** | **Catalog** | **Application** |
| --- | --- | --- | --- | --- |
| **WB** | ACSL4 | Abclonal | A20414 | 1:1000 |
|  | CD71 | Abclonal | A22161 | 1:3000 |
|  | CTGF | ProteinTech | 25474-1-AP | 1:1000 |
|  | DDR2 | Abcam | ab280354 | 1:1000 |
|  | GAPDH | ABclonal | AC001 | 1:5000 |
|  | GPX4 | ABclonal | A25009 | 1:1000 |
|  | Histone 3 | CST | 9715 | 1:1000 |
|  | SRC | Abcam | ab109381 | 1:1000 |
|  | Ubiquitin | CST | 3936 | 1:1000 |
|  | WWTR1 | ProteinTech | 23306-1-AP | 1:1000 |
|  | YAP1 | CST | 14074 | 1:1000 |
| **IP** | DDR2 | Abcam | 3B11E4 | 1:100 |
|  | GPX4 | ProteinTech | 67763-1-Ig | 1:100 |
|  | SRC | CST | 2110 | 1:50 |
|  | Mouse normal IgG | Beyotime | A7028 | 1:100 |
|  | Rabbit normal IgG | Beyotime | A7016 | 1:100 |
| **IF** | WWTR1 | ProteinTech | 23306-1-AP | 1:200 |
|  | YAP1 | CST | 14074 | 1:200 |
