# Supplementary figures and images for: Enhanced ferroptosis sensitivity promotes the formation of highly myopic cataract via the DDR2-Hippo pathway
Source: Cell Death Dis. 2025 Feb 3;16(1):64. doi: 10.1038/s41419-025-07384-8 (PMC11790942; doi:10.1038/s41419-025-07384-8)

**Fig. 2e**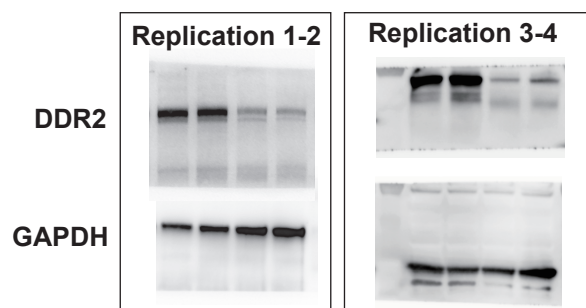**Fig. 2g**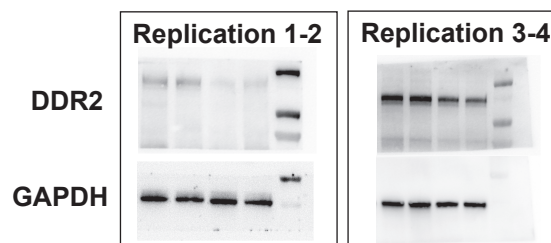**Fig. 3b**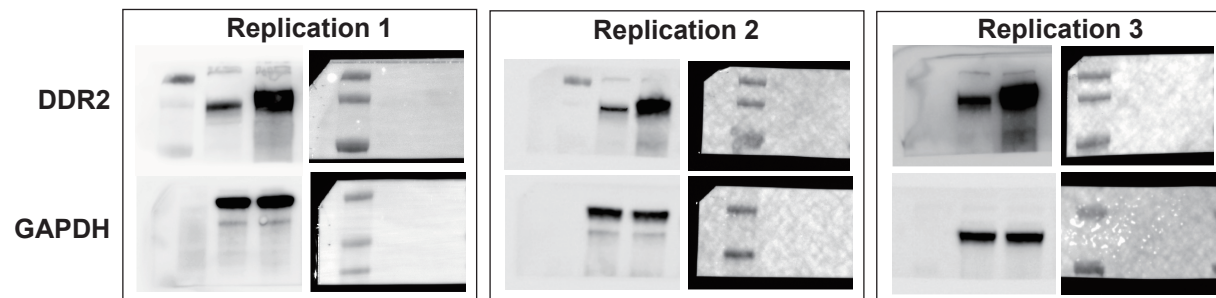**Fig. 4b**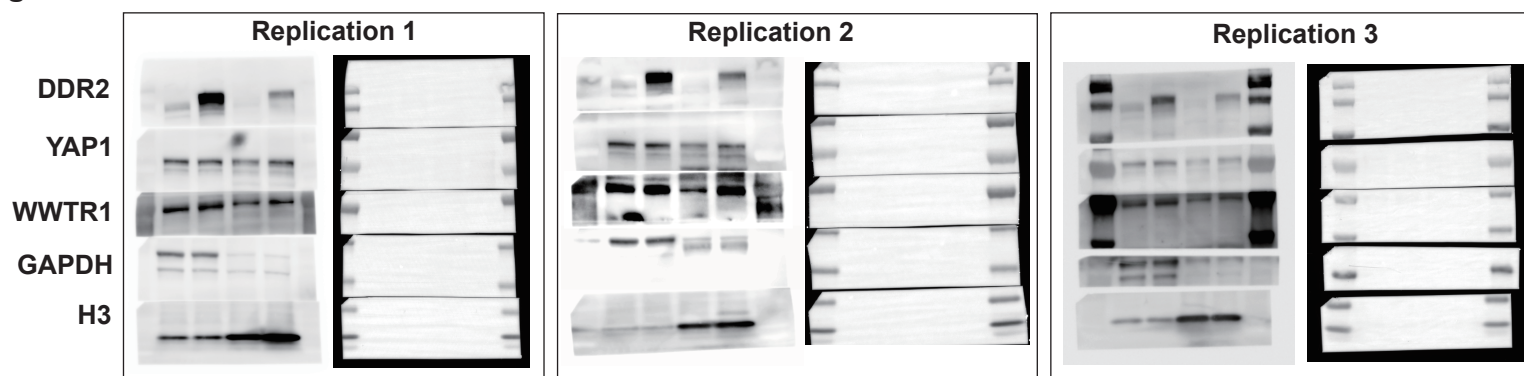**Fig. 4h**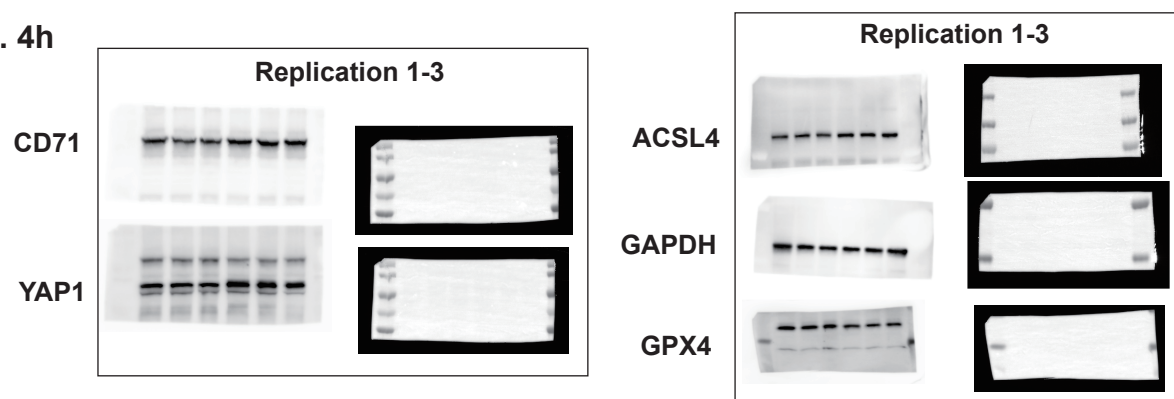**Fig. 4k**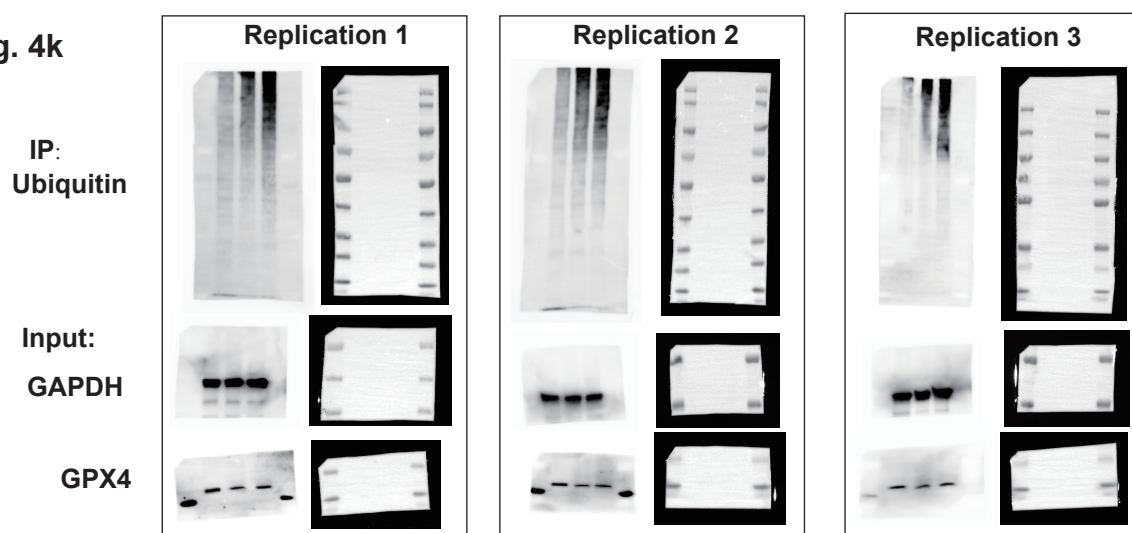

**Fig. 5b**

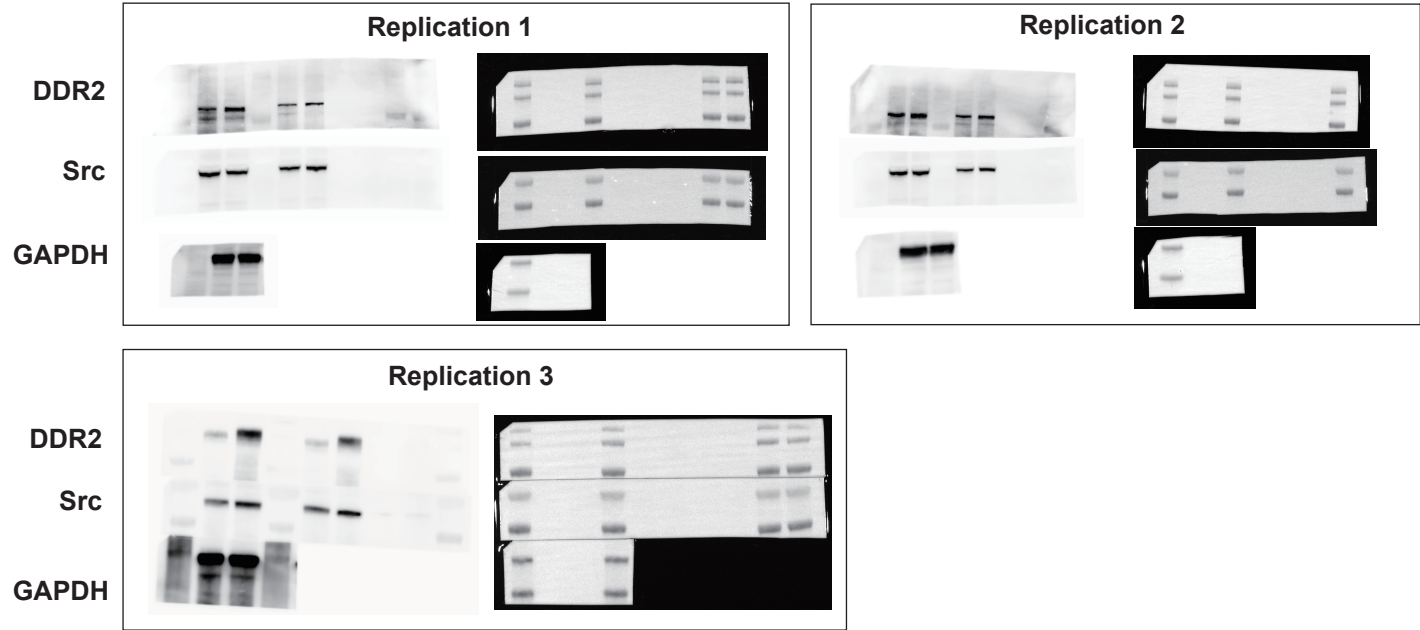

**Fig. S2a**

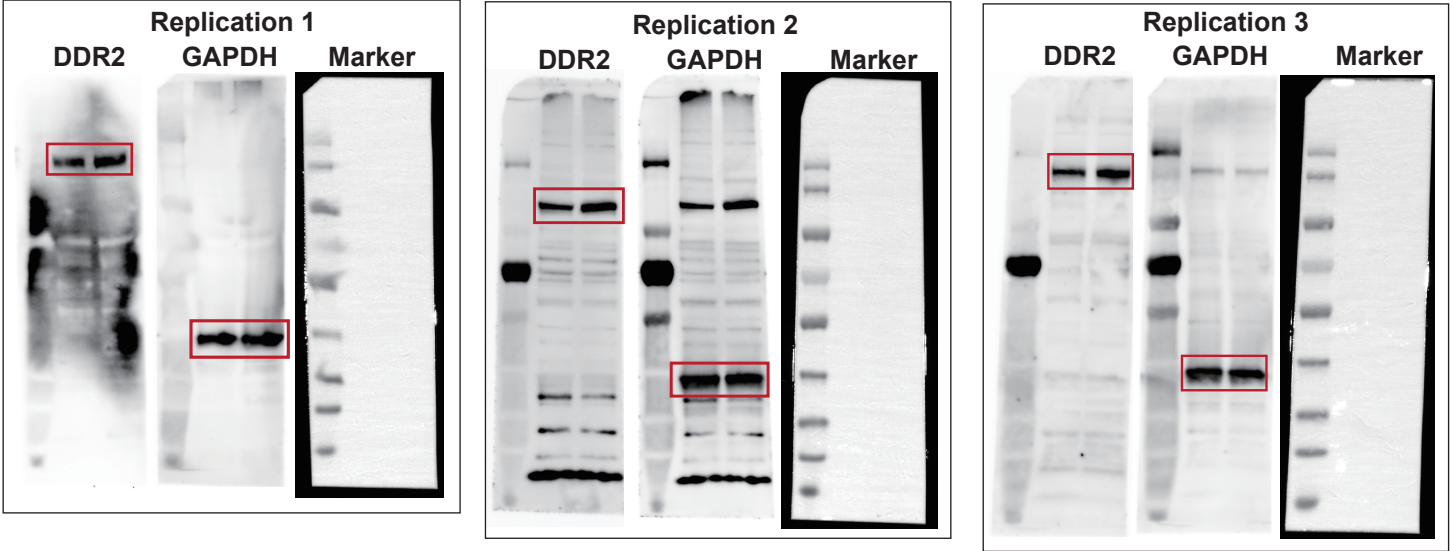

**Fig. S4d**

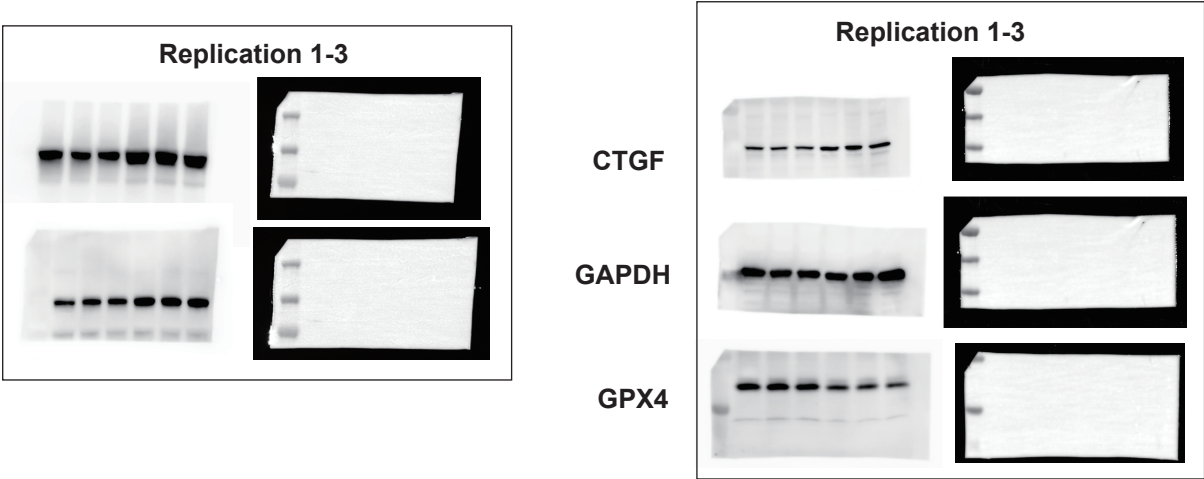

**Fig. S4f**

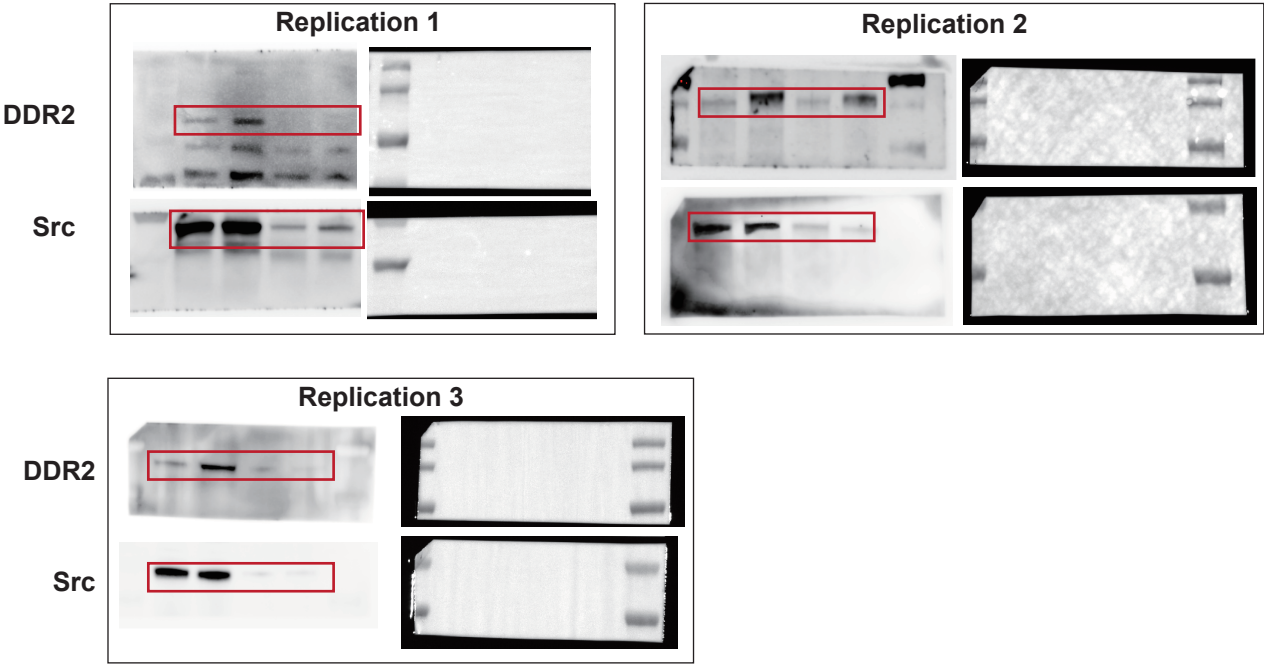

**Fig. S4h**

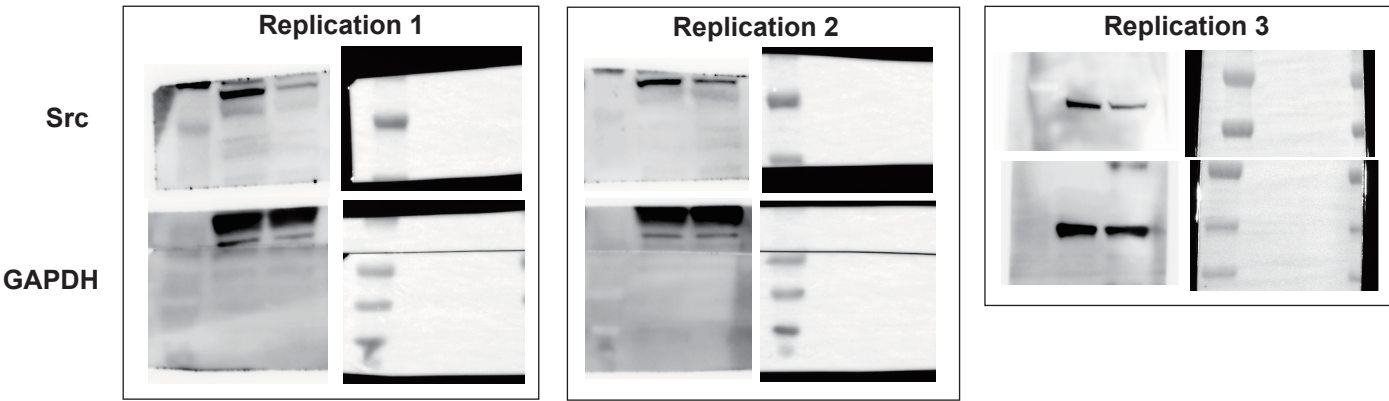

Fig. 1j

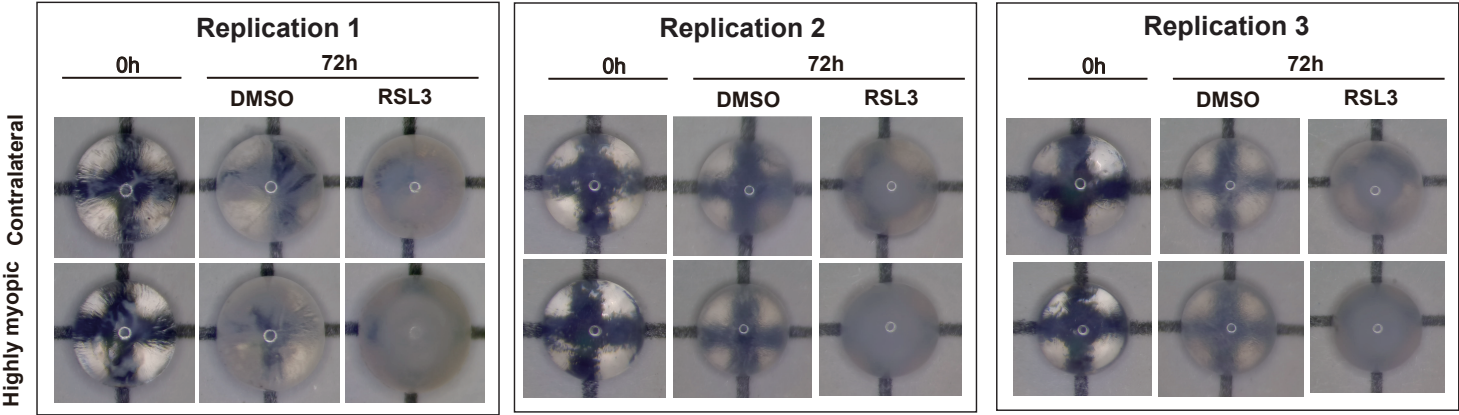

Fig. 6c

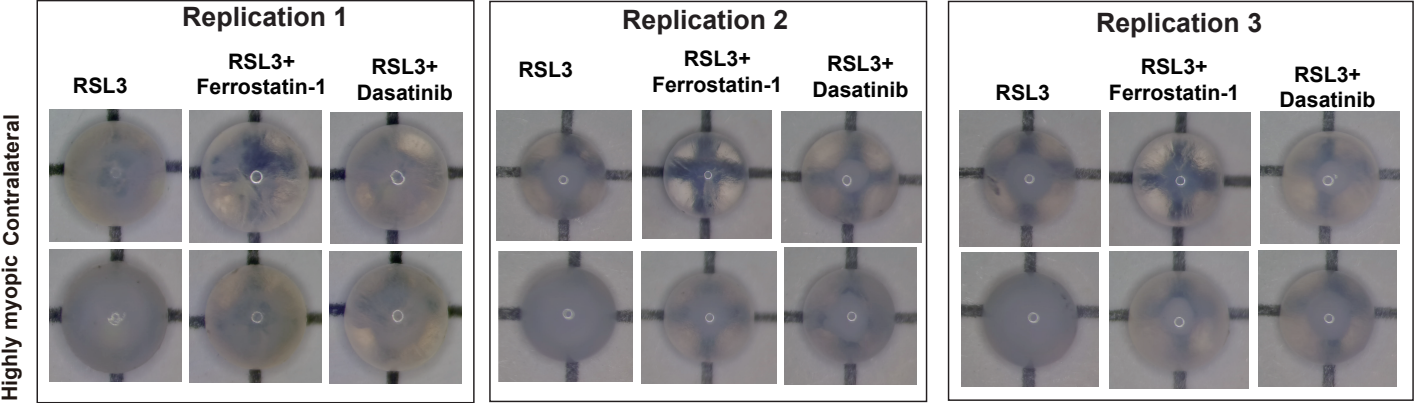

Fig. 6d

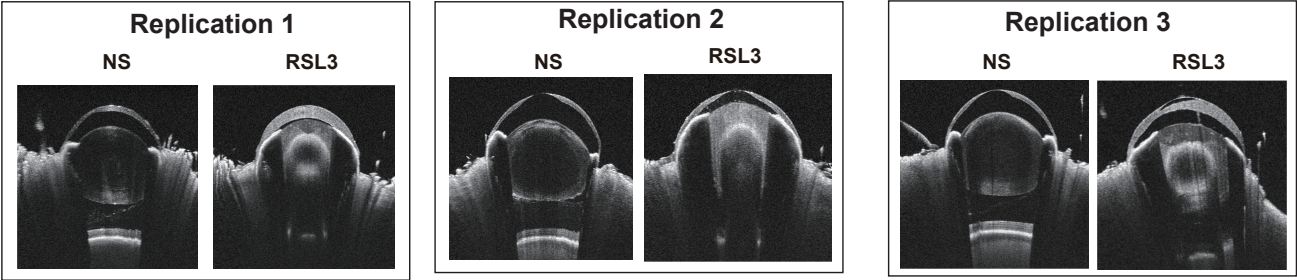

Fig. 6f

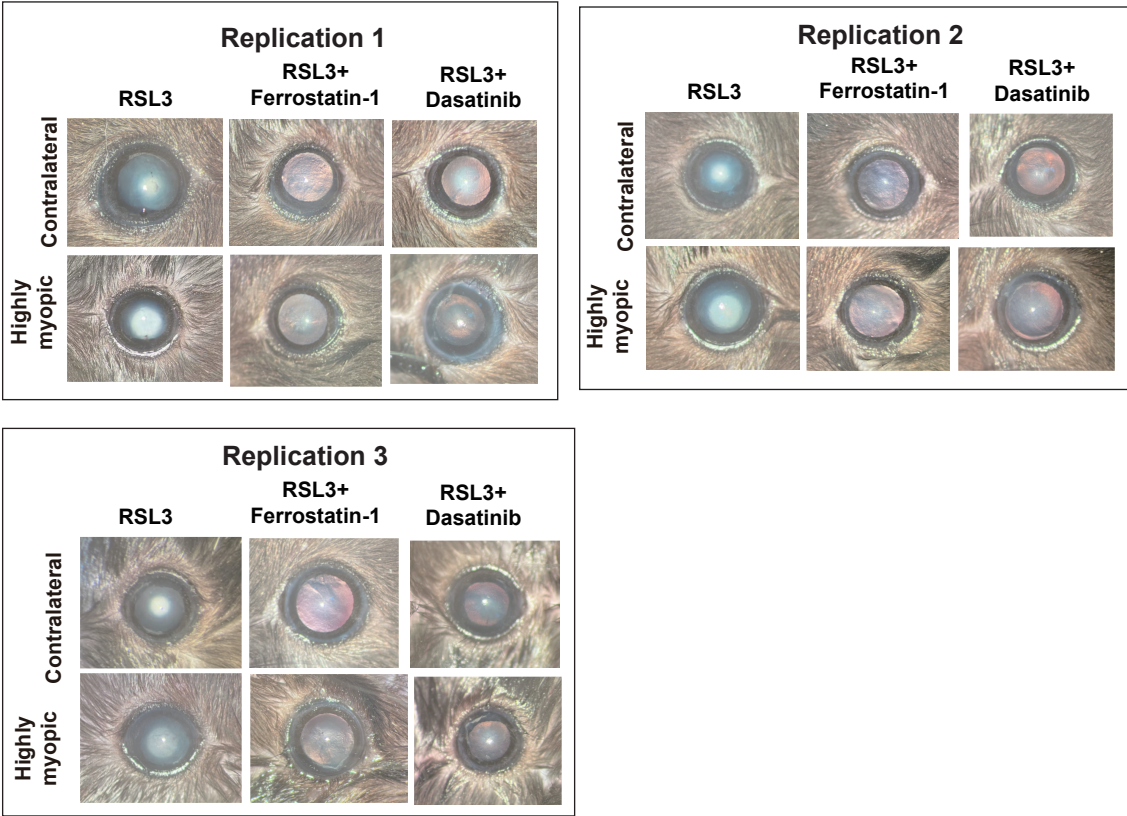

Supplement: Supplementary file 3 — Original data file [file 41419_2025_7384_MOESM3_ESM.pdf]
